# Human Adipose‐Derived Stem Cells Expanded Under Ambient Oxygen Concentration Accumulate Oxidative DNA Lesions and Experience Procarcinogenic DNA Replication Stress

**DOI:** 10.5966/sctm.2015-0401

**Published:** 2016-08-02

**Authors:** Rémy Bétous, Marie‐Laure Renoud, Claire Hoede, Ignacio Gonzalez, Natalie Jones, Michel Longy, Luc Sensebé, Christophe Cazaux, Jean‐Sébastien Hoffmann

**Affiliations:** ^1^ Equipe Labellisée La Ligue Contre Le Cancer, Paris, France; ^2^ Laboratoire d'Excellence Toulouse Cancer Labex Toucan, Cancer Research Center of Toulouse, INSERM U1037, CNRS ERL5294, Toulouse, France; ^3^ University Paul Sabatier, Toulouse, France; ^4^ Etablissement Français du Sang Pyrénées Méditerranée, Toulouse, France; ^5^ INSERM U1031, UMR5273, Toulouse, France; ^6^ Institut National de la Recherche Agronomique (INRA), UR 875, Unité de Mathématique et Informatique Appliquées, PF Bioinfo Genotoul, Castanet Tolosan, France; ^7^ INSERM U916 Vinco, Université de Bordeaux, Institut Bergonié, Bordeaux, France

**Keywords:** Human adipose‐derived stem cells, Ex vivo expansion, Oxygen concentration, DNA replication stress, DNA damage

## Abstract

Adipose‐derived stem cells (ADSCs) have led to growing interest in cell‐based therapy because they can be easily harvested from an abundant tissue. ADSCs must be expanded in vitro before transplantation. This essential step causes concerns about the safety of adult stem cells in terms of potential transformation. Tumorigenesis is driven in its earliest step by DNA replication stress, which is characterized by the accumulation of stalled DNA replication forks and activation of the DNA damage response. Thus, to evaluate the safety of ADSCs during ex vivo expansion, we monitored DNA replication under atmospheric (21%) or physiologic (1%) oxygen concentration. Here, by combining immunofluorescence and DNA combing, we show that ADSCs cultured under 21% oxygen accumulate endogenous oxidative DNA lesions, which interfere with DNA replication by increasing fork stalling events, thereby leading to incomplete DNA replication and fork collapse. Moreover, we found by RNA sequencing (RNA‐seq) that culture of ADSCs under atmospheric oxygen concentration leads to misexpression of cell cycle and DNA replication genes, which could contribute to DNA replication stress. Finally, analysis of acquired small nucleotide polymorphism shows that expansion of ADSCs under 21% oxygen induces a mutational bias toward deleterious transversions. Overall, our results suggest that expanding ADSCs at a low oxygen concentration could reduce the risk for DNA replication stress‐associated transformation, as occurs in neoplastic tissues. Stem Cells Translational Medicine *2017;6:68–76*

**Significance Statement**

The present work explored the influence of oxygen concentration on the induction of DNA replication stress, a major driver of tumorigenesis, during ex vivo amplification of adipose‐derived stem cells (ADSCs) used for cellular therapies. The currently used good manufacturing practices atmospheric oxygen condition (21%) compared with the physiologic one (1%) increased oxidative DNA damage and perturbed DNA replication by blocking DNA replication forks and, as a result, enhanced transversion mutations and activated the DNA damage response pathways. Collectively, these results strongly suggest that decreasing oxygen concentration could improve the safety of ADSCs during ex vivo expansion.

## Introduction

Human mesenchymal stem cells (hMSCs) are adult immature cells characterized by their self‐renewal ability and multipotency. First identified in bone marrow, MSCs found in the stromal vascular fraction of fat tissue have attracted growing interest in the past few years. Indeed, these adipose‐derived stem cells (ADSCs) can be isolated with minimal invasiveness by liposuction or dermolipectomy 1. Moreover, ADSCs provide tremendous advantages for regenerative medicine because they can be induced in vitro to differentiate or even transdifferentiate into several cell lineages 2. ADSCs can also home to damaged tissues and enhance tissue repair by secreting angiogenic, antiapoptotic, immunomodulatory, and hematopoietic factors 2. Thus, ADSCs represent an attractive material for cell‐based therapies 2.

Even if ADSCs represent as much as 1% of adipose cells, the quantity obtained when extracted from a donor is far less than the required number of hMSCs currently needed for clinical trials (typically 50–200 million hMSCs) 3 4 5. Thus, hMSCs, such as ADSCs, must be expanded in vitro before transplantation. This essential step has raised important concerns about the safety of adult stem cells. Two studies have reported transformation of hMSCs after long‐term culture expansion, but these articles were subsequently retracted because of cross‐contamination by an exogenous cancer cell line 6, 7. However, a recent study detected spontaneous transformation of hMSCs with cancer‐associated genetic modifications during culture expansion 8. In this latter report, the authors excluded the possibility of human cell line contamination, confirming the concerns about the safety of hMSCs. There is therefore an urgent need to fine‐tune hMSC culture methods to prevent or at least limit the risk for hMSC transformation during in vitro expansion.

hMSCs are currently expanded under an atmospheric oxygen concentration and are therefore exposed to 21% oxygen, which is approximately 2–20 times more than the physiological oxygen concentration found in different organs. Importantly, two studies have reported that a low oxygen concentration during MSC expansion limits genetic instability, a well‐known driver of tumorigenesis 9, 10. The authors observed less aneuploidy and chromosomal aberrations in both mouse and human ADSCs cultured under 2% or 3% oxygen compared with those cultured under an atmospheric oxygen concentration. Such instability, mainly resulting from errors during the course of genome duplication (so‐called replicative stress), is now accepted as contributing to the emergence and progression of cancer, even during the early stages of tumor development 11. Thus, to prevent cellular transformation during ex vivo expansion of hMSCs, culture conditions should limit DNA replication stress. The present study aims to address whether oxygen concentration affects DNA replication during the first few cell passages that follow extraction of adult ADSCs from healthy donors, and thus whether the induction of potential cancer‐promoting genetic changes in vitro is oxygen concentration‐dependent.

## Materials and Methods

### Isolation and Culture of ADSCs

Three clinical‐grade productions of ADSCs, from three different donors, were cultured from passage (P) 1 to P12. ADSCs were isolated from subcutaneous adipose tissue obtained from nonobese patients (body mass index, 27.08–27.7) undergoing elective abdominal dermolipectomy (Plastic Surgery Department, Rangueil Hospital, Toulouse, France). No objection certificates were obtained according to the bioethics law no. 2004‐800 of August 6, 2004. Stromal vascular fraction (SVF) was obtained by digestion of adipose tissue with collagenase NB4. SVF cells were plated at 4,000 cells per cm^2^ and cultivated in α‐minimal essential medium supplemented with 2% platelet lysate, 1% penicillin/streptomycin (Thermo Fisher Scientific Life Sciences, Waltham, MA, http://www.thermofisher.com), 0.1% amphotericin B (Thermo Fisher), 1 U/ml heparin, at 37°C. Medium was changed twice a week. Cells were cultured under normoxic (20% O_2_, 5% CO_2_) or hypoxic (1% O_2_, 5% CO_2_) conditions in an Xvivo System (BioSpherix, Paris, NY, http://www.biospherix.com) for maintaining cells in hypoxic condition at all culture steps. Cells were harvested at 80% confluence, then counted and seeded at 2,000 cells per cm^2^ for the following passages (P1–P12).

### Cell Cycle Analysis

ADSCs were seeded at a density of 3,000 cells per cm^2^. After 72 hours, cells were harvested and fixed in 70% ethanol for 2 hours on ice, washed with phosphate‐buffered saline (PBS), and incubated in PBS‐0.1% Tween containing 25 µg/ml propidium iodide and 250 µg/ml RNase‐A for 15 minutes. Cells were analyzed with a FACSCalibur system (BD Biosciences, Franklin Lakes, NJ, http://www.bdbiosciences.com). Cell cycle repartition was assessed with Modfit LT 4.0 (Verity Software House, Topsham, ME, http://www.vsh.com).

### Antibodies and Immunofluorescence

ADSCs were seeded on glass coverslips at a density of 3,000 cells per cm^2^ in 6‐well plates. Seventy‐two hours later, cells were fixed for 10 minutes in 4% paraformaldehyde (PAF) at room temperature (RT) for p53‐binding protein 1 (53BP1) and cyclin‐A detection or pre‐extracted for 30 seconds in SDS buffer (20 mM Hepes, pH 7.4, 560 mM NaCl, 0.1% Triton X‐100, and 0.02% SDS) on ice for 7,8‐dihydro‐8‐oxoguanine (8‐oxoG) detection before PAF fixation. Cells were then washed 3 times with PBS and permeabilized for 10 minutes on ice in permeabilization buffer (20 mM Hepes, pH7.4, 50 mM NaCl, 3 mM MgCl_2_, 300 mM sucrose, and 0.5% Triton X‐100). After 3 washed in PBS, coverslips were blocked in PBS‐5% bovine serum albumin for 15 minutes at RT. Cells were incubated with primary antibodies against 8‐oxoG (1:150, SC‐130914; Santa Cruz Biotechnology, http://www.scbt.com/) or 53BP1 (1:200, ab21083; Abcam, Cambridge, UK, http://www.abcam.com) and cyclin‐A (1:150, GTX73860; GeneTex, Irvine, CA, http://www.genetex.com) for 1 hour at RT in PBS and then incubated with Alexa Fluor 488 or 555 goat anti‐mouse or anti‐rabbit antibodies (1:1,000; Molecular Probes, Thermo Fisher) for 1 hour at RT in PBS. Coverslips were mounted in ProLong Gold antifade reagent (ThermoFisher). Image acquisition of multiple random fields was performed on a wide‐field microscope (model DMLA, Leica Biosystems, Wetzlar, Germany, http://www2.leicabiosystems.com) equipped with ×63, type PL, NA 1.25 objective and using a charge coupled device camera (CoolSNAP HQ, Photometrics, Tucson, AZ, http://www.photometrics.com/) driven by PM Capture Pro 6.0 (Photometrics). For 53BP1 nuclear bodies, the cyclin‐A‐negative nuclei were scored for the presence of 53BP1 large foci. For 8‐oxoG measurement, fluorescence intensity was quantitated with Cell Profiler software (Broad Institute, Cambridge, MA, http://www.cellprofiler.org).

### Alkaline Comet Assay

An alkaline comet assay was performed in accordance with the manufacturer's (CometAssay, Trevigen, Gaithersburg, MD, https://www.trevigen.com) instructions.

### Nucleic Acid Extraction

ADSC DNA and RNAs were extracted from 2 million cells by using, respectively, the Qiagen DNeasy Blood and Tissue and the Qiagen RNeasy extraction kits according to the manufacturer's instructions (Qiagen, Hilden, Germany, https://www.qiagen.com).

### Comparative Genomic Hybridization Array

ADSC DNA (500 ng) and a reference pool of female DNA (500 ng; Promega Human Genomic DNA: Female G1521; Promega, Madison, WI, http://www.promega.com) were labeled with Cy5‐2′‐deoxyuridine 5′‐triphosphate (dUTP) and Cy3‐dUTP, respectively, by using the SureTag DNA Labeling Kit according to the manufacturer's instructions (Agilent Technologies, Santa Clara, CA, http://www.agilent.com). The labeled DNA was hybridized to a SurePrint G3 Human 4 × 180K comparative genomic hybridization (CGH) microarray with an overall median probe spacing of 13 kb (11 kb in Reference Sequence genes) and washed according to the manufacturer's instructions (Agilent Technologies). Arrays were scanned by using an Agilent G2565CA microarray scanner; images were analyzed by using Feature Extraction software, version 10.1.1.1; and data analysis was performed by using Genomic Workbench 7.0 (Agilent Technologies). ADSC DNA from donors 1, 2, and 3 was cultured at 1% and 21% O_2_; extracted at P1, P4, and P10; and analyzed by CGH array. Their genomic profiles were compared to identify culture‐induced chromosomal aberrations.

### RNA Sequencing

RNA sequencing (RNA‐seq) was performed at the GeT‐PlaGe core facility, INRA Toulouse, Toulouse, France. RNA‐seq libraries have been prepared according to Illumina's protocols on a Tecan EVO200 liquid handler using the Illumina TruSeq Stranded mRNA sample prep kit to analyze mRNA (Tecan, Männedorf, Switzerland, http://lifesciences.tecan.com). Briefly, mRNA was selected by using poly‐T beads. Then, RNA was fragmented to generate double‐stranded cDNA and adaptors were ligated to be sequenced. Ten cycles of polymerase chain reaction (PCR) were applied to amplify libraries. Library quality was assessed by using an Agilent Bioanalyzer (Agilant Biotechnologies, Santa Clara, CA, http://www.genomics.agilent.com) and libraries were quantified by quantitative PCR using the Kapa Library Quantification Kit (Kapa Biosystems, Wilmington, MA, https://www.kapabiosystems.com). RNA‐seq experiments were performed on an Illumina HiSeq2000 using a paired‐end read length of 2 × 100 bp with the Illumina TruSeq SBS sequencing kit, version 3. We performed spliced mapping with the Spliced Transcripts Alignment to a Reference (STAR) software, version 2.3.1f 12, against *Homo sapiens* genome (Ensembl 37.74, http://useast.ensembl.org). Then we sorted, removed duplicates from alignment files, and merged them. We used Cufflinks software, version 2.1.1 (University of Washington, Seattle, WA, http://cole-trapnell-lab.github.io/cufflinks/) 13 to build new transcripts and merge the annotation file with the reference annotation file from Ensembl ( http://useast.ensembl.org). With a homemade script, we computed raw reads counts on each transcripts/genes and renamed genes with Ensembl id.

### Differential Expression Analysis

Raw count RNA‐seq data at the gene level (three replicates for each growth condition with, respectively, 1% and 21% O_2_ in the medium to P1) were processed and analyzed within the R computing environment ( https://www.r-project.org) 14 by using the edgeR package 15 from Bioconductor ( https://bioconductor.org). Raw data were normalized by using the trimmed mean of M‐values normalization procedure 16 to account for the different sequencing depths between the samples. To deal with very lowly expressed genes in any of the experimental conditions, normalized data were submitted to filtering. We filtered out genes that were not expressed and kept genes that were expressed in at least one sample. The relationships between conditions and library reproducibility within each condition were determined by a multidimensional scaling method. Differential gene expression was evaluated by using an overdispersed Poisson (negative binomial) model combined with a likelihood ratio test 17. In a manner analogous to a one‐way analysis of variance test, we performed differential expression analysis of count data between the two groups: hypoxia vs. normoxia. Because the samples come from three different donors receiving both hypoxia and normoxia treatment, a paired design was used. The design was formed from an additive model that included both the donor and the group factor without the interaction term. Genes with significant evidence for differential expression were evaluated by an adjusted *p* value based on the Benjamini‐Hochberg multiple testing correction 18 in conjunction with a data‐based filtering procedure using the HTSFilter 19 package of Bioconductor. Adjusted *p* value and fold change thresholds were used to determine differentially expressed genes.

### Pathway Enrichment Analysis

EntrezGene ID of differentially expressed genes were entered into the WebGestalt resource ( http://bioinfo.vanderbilt.edu/webgestalt/). For analysis, the following parameters were entered: reference, *Homo sapiens* genome; method, hypergeometric; multiple test adjustment, Benjamini‐Hochberg; significance level, .05; database, Kyoto Encyclopedia of Genes and Genomes (KEGG) pathway ( http://www.genome.jp/kegg/pathway.html)

### Identification of Small Nucleotide Polymorphisms

We added read groups in STAR alignment files, merged them, and removed multimapped reads. The mapping quality was set to 40 for all uniquely mapped reads. We used the GATK software package, version 3.0 (Broad Institute) 20, 21 to perform all the variant calling by following the best practices recommendations for variant calling in RNA‐seq data. We used the GATK tools for split spliced reads and performed the indel (insertion and deletion) realignment with the indel Mills and 1KG databanks. We recalibrated base quality with dbSNP software, version 138 (National Center for Biotechnology Information, Bethesda, MD, http://www.ncbi.nlm.nih.gov) 22 and previous indel databanks. Then, we used HaplotypeCaller (Broad Institute) to detect small nucleotide polymorphisms (SNPs) and indels. Finally, we filtered variants if there were more than 3 variants in a 35‐bp window, if their strand bias was greater than 30, and if quality normalized by depth was less than 2.0. We used SnpEff 23 to functionally annotate the variants and SnpSift ( http://snpeff.sourceforge.net/ for both packages) 24 to obtain dbSNP id.

### DNA Combing

DNA combing experiments were carried out as described elsewhere 25.

## Results

### ADSCs Cultured Under 21% Oxygen Accumulate Oxidative DNA Lesions

**Figure 1:**
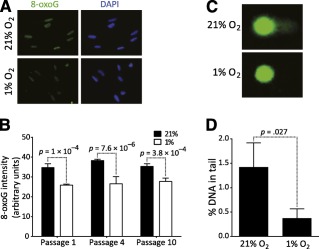
Adipose‐derived stem cells (ADSCs) cultured under 21% O_2_ accumulate oxidative DNA lesions. **(A):** Representative image of ADSCs immunostained with an anti‐8‐oxoG antibody. **(B):** Quantification of 8‐oxoG signal in nuclei of ADSCs expanded under 21% or 1% oxygen. **(C):** Representative image of cells subjected to alkaline comet assay. **(D):** Quantification of DNA in comet tail after alkaline comet assay. Error bars represent SD from three independent donors, and significance was determined by *t* test. Original magnification, ×63. Abbreviations: DAPI, 4′,6‐diamidino‐2‐phenylindole; 8‐oxoG, 7,8‐dihydro‐8‐oxoguanine.

In vivo MSCs reside in an environment exposed to a physiological oxygen concentration ranging from 0.5% to 14%, depending on the organ 26. Expanding MSCs ex vivo in an atmospheric condition induces oxidative stress 9, 27 and higher levels of intracellular reactive oxygen species (ROS) than those cultured under 5% oxygen 27. Besides the resulting increase in protein and lipid oxidation 9, 27, it is widely reported that such oxidative stress can also generate more than 20 different types of DNA modifications 28. We therefore investigated the influence of oxygen concentration on the most abundant and mutagenic oxidative DNA lesion (8‐oxoG), commonly used as a biomarker for oxidative DNA damage, in cultures of ADSCs from three donors and at three different cell passages (P1, P4, P10). Stem cells were exposed to 1% oxygen (hypoxia) or 21% (normoxia) oxygen, the atmospheric culture condition. We evaluated the abundance of this DNA lesion in ADSCs cultured in a hypoxic or normoxic environment by immunofluorescence by using an 8‐oxoG‐specific antibody. As presented in Figure 1A and 1B, we found that ADSCs expanded under 21% oxygen contain significantly more 8‐oxoG than ADSCs cultured under 1% oxygen, suggesting that ADSCs accumulate oxidative DNA lesions during ex vivo expansion under an atmospheric oxygen concentration.

Because ROS can directly produce single‐ or double‐stranded DNA breaks as well as alkali labile DNA lesions, we then performed a comet assay under alkaline conditions to quantify such clastogenic events. Again, we found a higher DNA content in the comet tails of ADSCs cultured in the normoxic condition than those cultured in the hypoxic condition (Fig. 1C, 1D). Overall, these results confirm that ADSCs cultured under 21% oxygen accumulate oxidative DNA lesions.

### ADSCs Cultured Under 21% Oxygen Experience DNA Replication Stress

**Figure 2:**
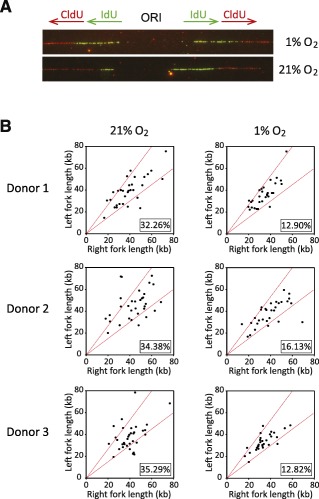
Culture of adipose‐derived stem cells (ADSCs) under 21% oxygen shows increased DNA replication fork stalling. **(A):** Example of symmetric (1%) and asymmetric (21%) sister forks. IdU and CldU tracks are shown, as well as the ORI. **(B):** Scatter plots of the distances traveled by right‐moving and left‐moving sister forks during the IdU pulse in ADSCs. The central areas delimited with red lines contain sister forks with less than 25% length difference. The percentages of outliers (asymmetric sister forks) are indicated (lower right of plots). Abbreviations: CldU, chloro‐deoxyuridine; IdU, iodo‐deoxyuridine; ORI, origin of replication.

DNA replication is an essential process that is tightly regulated to ensure genome stability. It is initiated from tens of thousands of replication origins dispersed throughout the genome. From these origins emanate diverging sister replication forks that replicate hundreds to thousands of kilobases of DNA. During this process, replication forks encounter many obstacles that block the replicative DNA polymerases and induce fork stalling. Such obstacles include DNA‐bound proteins and DNA secondary structures but also unrepaired DNA damage 29. Importantly, accumulation of stalled DNA replication forks defines replicative stress, which is strongly associated with chromosomal instability in cancer cells 30. We therefore investigated whether oxidative DNA lesions generated during ADSC expansion in the normoxic condition could alter the progression of the replisome machinery. We carried out DNA combing experiments both at the whole genome and single DNA molecule levels. ADSCs were incubated for 20 minutes in the presence of the nucleotide analogs iodo‐deoxyuridine and chloro‐deoxyuridine before DNA extraction, combing, and immunodetection (Fig. 2A). To estimate the proportion of stalled DNA replication forks, we compared the length of the sister forks diverging bidirectionally from the same replication origin. If a replication fork stalls, the length of the replication track synthesized by this fork during nucleotide analog incorporation will be shorter than the track synthesized by the corresponding and opposite sister fork. We considered as asymmetric the sister forks with length difference more than 25%. We found that ADSCs expanded under 21% oxygen have more asymmetric sister forks than ADSCs cultured under 1% oxygen (Fig. 2A, 2B and supplemental online Fig. 1A).

**Figure 3:**
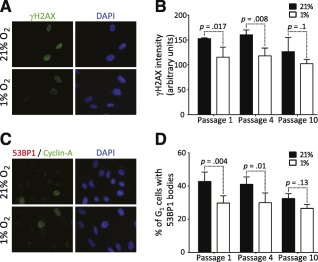
Adipose‐derived stem cells (ADSCs) grown under high oxygen tension exhibit manifestations of replicative stress. **(A):** Representative image of ADSCs immunostained with an anti‐γH2AX antibody. **(B):** Quantification of γH2AX signal in nuclei of ADSCs expanded under 21% or 1% oxygen. **(C):** Representative image of ADSCs immunostained with anti‐53BP1 and anti‐cyclin‐A antibodies. **(D):** Quantification cyclin‐A‐negative cells containing large 53BP1 bodies. Error bars represent SD from three independent donors, and significance was determined by *t* test. Original magnification,×63. Abbreviations: 53BP1, p53‐binding protein 1; DAPI, 4′,6‐diamidino‐2‐phenylindole.

To confirm these data, we first quantified the amount of the phosphorylated form of the histone variant H2AX (γH2AX) by immunofluorescence. Indeed, H2AX is phosphorylated at stalled replication forks by the kinase ataxia telangiectasia mutated and Rad3‐related (ATR) protein, the replication pause sensor; therefore, γH2AX is widely used as a marker of DNA replication stress. As expected, we observed higher γH2AX signals in the nuclei of ADSCs cultured in the normoxic condition at P1 and P4 (Fig. 3A, 3B). At P10 in the normoxic condition, the decrease of γH2AX signal, which is S phase‐dependent, probably reflects a decrease of cycling cells and senescence ( supplemental online Fig. 2).

Recently, it has been reported that under‐replicated DNA due to replication stress can be transmitted as broken DNA to daughter cells 31, 32. These DNA lesions are shielded by the repair protein 53BP1, which forms large nuclear bodies around such lesions in G_1_‐phase daughter cells 31, 32. We found that the proportion of cyclin‐A‐negative (G_1_‐phase) cells with large 53BP1 bodies was higher in ADSCs expanded under 21% oxygen than in ADSCs expanded under 1% oxygen at P1 and P4 (Fig. 3C, 3D), suggesting that ADSCs cultured in normoxia accumulate under‐replicated loci. As for the γH2AX signals, no significant difference in 53BP1 body formation was observed at P10, reflecting again the decrease of cycling cells (Fig. 3D and supplemental online Fig. 2). Collectively, these data indicate that ADSCs cultured under 21% oxygen exhibit increased DNA replication stress, which can be transmitted to daughter cells during ex vivo culture.

### Misexpression of Cell Cycle and DNA Replication Genes in ADSCs Switched to Normoxic Condition

Next, we explored whether deregulated gene expression in atmospheric oxygen condition might also contribute to the replication problems detected in these cells by performing RNA‐seq on ADSCs harvested at P1. We identified 747 genes whose expression significantly differed between the two cell culture conditions with a false discovery rate (FDR)‐adjusted *q*‐value < 0.050 ( supplemental online Table 1). A total of 310 genes were upregulated and 437 were downregulated in cells expanded under 21% oxygen. We then identified metabolic pathways enriched in this gene list. Fifty‐three pathways were significantly enriched, with an FDR‐adjusted *q*‐value < 0.050 ( supplemental online Table 2). As previously described 9, glycolysis appears in the top 5 enriched pathways (Table 1). We found that genes involved in cell cycle control and purine metabolism pathways that have critical regulatory and housekeeping functions during DNA replication were also highly enriched. Importantly, under 21% oxygen, we also discovered defective expression of critical DNA replication genes encoding replicative DNA polymerases (*POLD* and *POLE*), replicative helicase (*MCM3*, *GINS3*), and replication initiation factors (*TOPBP1, GMNN*, and *DBF4*) (Table 2). Only *ORC4*, which is part of the origin recognition complex, was upregulated. The overall differential gene expression profile therefore showed that transcription of genes involved in genome duplication is highly affected by oxygen concentration during ADSC ex vivo expansion.

**Table 1:** Top five enriched pathways of differentially expressed genes between adipose‐derived stem cells expanded under 21% or 1% oxygen

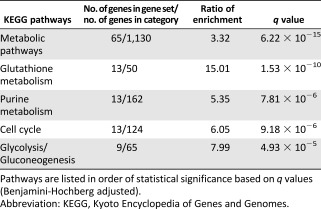

**Table 2:** Differentially expressed DNA replication genes between adipose‐derived stem cells cultured under 21% or 1% oxygen

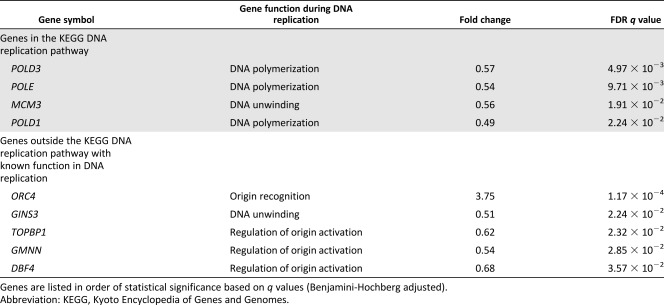

### Chromosome Structure of ADSCs Remains Stable Under 21% O_2_

It is now well‐established that replication stress induces different forms of genetic instability, including gross chromosomal instability or mutagenesis at the more subtle nucleotide level 11. To investigate whether the accumulation of stalled replication forks observed in cells cultured under an atmospheric oxygen concentration could induce chromosomal instability, we performed a CGH array. We observed almost identical genomic profiles from P1 to P10 for each donor, with the exception of ADSCs from donor 2 cultured under the hypoxic condition, which showed an amplification of chromosome 11 ( supplemental online Fig. 3). Karyotyping confirmed the presence of a clonal chromosome 11 trisomy from passage 7 (data not shown). Overall, we conclude that no significant genomic instability (as can be detected with the resolution of the CGH array) arose during ADSC ex vivo expansion, regardless of the culture conditions.

### Culture of ADSCs Under 21% Oxygen Induces a Transversion Mutation Bias

**Figure 4:**
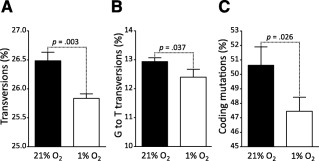
Bias toward transversion and coding mutations in adipose‐derived stem cells (ADSCs) cultured under 21% oxygen. Small nucleotide polymorphisms arising at passage 4 were classified. **(A):** Proportion of base transversions to total base substitutions. **(B):** Proportion of G‐to‐T transversions to total base transversions. **(C):** Proportion of coding mutations to total mutations. Error bars represent SD from three independent donors, and significance was determined by *t* test.

We next investigated whether the replication stress induced by higher oxygen concentration could qualitatively influence the DNA replication process. We monitored the appearance of SNPs by RNA‐seq at P4 on ADSCs from the three different donors. We considered as a point mutation any SNP occurring at P4 that was not present or sequenced at passageP1. We found that ADSCs expanded under the normoxic condition showed a higher proportion of transversions than cells cultured under 1% oxygen (Fig. 4A). Among the additional mutations, the proportion of G‐to‐T transversions was significantly higher in ADSCs expanded under 21% oxygen (Fig. 4B). Moreover, coding mutations were enriched in ADSCs cultured under the normoxic condition (Fig. 4C), suggesting that expansion of mesenchymal stem cells under 21% oxygen could affect protein functions through deleterious mutations of genes. Taken together, these results suggest that a subtle mutational bias at the nucleotide level toward nucleotide transversion could impair genome stability of ADSCs cultured under 21% O_2_.

## Discussion

Good manufacturing practices (GMP) for hMSC‐based cellular therapy vary among laboratories, with no internationally recognized standard practice. A key challenge for the next few years is the standardize GMP to achieve safe and efficient hMSC expansion before transplantation. Recently, efforts have been made toward improving media composition. Platelet lysate has emerged as a better alternative to fetal bovine serum (FBS) to achieve a faster proliferation rate and improved chromosomal stability 33, 34. Accumulating evidence also suggests that oxygen concentration strongly influences hMSC behavior in culture 35. Lowering the oxygen concentration could also enhance hMSC proliferation with concomitant limitation of genetic instability 9. However, the underlying molecular mechanisms have not yet been identified. Here, we show that ADSCs cultured under 21% oxygen accumulate endogenous oxidative DNA lesions which interfere with DNA replication by increasing fork stalling events, leading to incomplete DNA replication and fork collapse. Moreover, culture of ADSCs under an atmospheric oxygen concentration may induce a mutational bias toward deleterious transversions. Our results also suggest that these effects could be potentiated by the repression of DNA replication gene expression.

It has been demonstrated that cancer risk is strongly correlated with the total number of stem cell division in tissues, increasing the lifetime risk for cancer 36. Moreover, it is well recognized that genomic instability is a hallmark of cancer, generating the genetic diversity that shapes tumor development 37. Evidence suggests that genomic instability of cancer cells is a consequence of replicative stress, through the accumulation of stalled replication forks during the earliest stages of tumorigenesis 11. To obtain the relevant number of cells currently required for clinical trials, hMSCs have to be expanded ex vivo and therefore divide multiple times before transplantation. To limit the risk for tumorigenic cell transplantation, culture conditions must therefore not alter DNA replication to prevent genetic instability at both nucleotide and chromosomal levels.

In this study, we hypothesized that the elevated oxygen concentration in current GMP protocols for hMSC expansion could induce the accumulation of oxidative DNA lesions, which could, in turn, alter DNA replication. We did indeed observe an increased level of 8‐oxoG in ADSC nuclei expanded under 21% oxygen. The similar levels of 8‐oxoG at different passages suggest both the absence of any compensation over time by repair pathways such as base excision repair and the presence of a constant DNA damage pressure on the genome of ADSCs expanded under the atmospheric condition. Oxidative stress processes not only 8‐oxoG but also at least 20 different types of base modifications, as well as single‐ and double‐strand breaks 28. Some of these lesions are alkali labile, and we found that expanding ADSCs under atmospheric oxygen concentration increases the production of alkali labile DNA lesions. Thus, ADSCs cultured under 21% oxygen exhibited increased oxidative DNA lesions compared with ADSCs cultured under 1% oxygen.

We reasoned that accumulation of oxidative DNA lesions could impede DNA replication, creating a state of replicative stress. Various definitions of replication stress have been proposed, but the accumulation of stalled replication forks is common to all of them. We used DNA combing to assess the proportion of stalled DNA replication forks during genome duplication. We found that sister forks were more asymmetric in cells cultured under the normoxic than those cultured under the hypoxic condition (Fig. 2A, 2B). We therefore concluded that ADSCs expanded under 21% oxygen experience DNA replication stress. Because DNA replication stress is now recognized as a strong driver of tumorigenesis 11, our results suggest that ADSCs cultured under atmospheric oxygen concentration are more likely to transform than cells cultivated under hypoxia. Human MSCs rarely transform in culture, making the direct evaluation of the effect of oxygen concentration on the promotion of tumorigenic cells during ex vivo expansion very difficult. However, growth under 5% oxygen prevents mouse MSC from undergoing spontaneous p53‐dependent transformation 27.

To confirm this important observation, we investigated DNA replication stress consequences at the molecular level. Stalled replication forks elicit an ATR‐dependent DNA damage response pathway that leads to the phosphorylation of the histone variant H2AX. They may eventually collapse and form DNA double‐strand breaks, also leading to the phosphorylation of H2AX in an ataxia telangiectasia mutated and DNA‐dependent protein kinase‐dependent manner. Finally, increased fork stalling events lead to the accumulation of under‐replicated regions that are transformed into DNA lesions during mitosis and transmitted to daughter cells, where they are shielded by large 53BP1 bodies 31, 32. We found that both H2AX phosphorylation and the proportion of daughter cells displaying large 53BP1 bodies were increased in cells expanded under the normoxic condition, confirming that these cells experience DNA replication stress. Our study therefore demonstrates the consequences of DNA replication stress on cancer‐promoting genomic instability when cells are cultured under 21% oxygen.

Our results clearly show that ADSCs expanded under atmospheric oxygen conditions are prone to chronic DNA replication stress and therefore to the activation of the DNA damage response, yet discrepancies exist in the literature concerning the consequences of hypoxia on genomic stability. Some studies reported that a low oxygen concentration induces microsatellite instability and chromosomal variability in hMSC 38, 39. In contrast, others have shown that expanding human or mouse MSCs under 3% or 2% oxygen decrease aneuploidy and chromosomal aberrations 9, 10, a finding corroborated by further studies performed with different cell types, such as mouse embryonic fibroblasts or human embryonic cells 40, 41.

Differences in MSC culture protocols may account for such contrasting results; in all studies using mesenchymal stem cells, the authors used media containing FBS to expand cells. In such media, cells grow much more slowly than in platelet lysate‐containing media, and there is a strong proliferation bias for hMSCs cultured under low oxygen concentration 9, 42 because they grow faster than normoxia‐cultivated cells. Mesenchymal stem cells expanded in the hypoxic condition therefore pass through S‐phase more often than those cultured under 21% oxygen, giving more opportunities to fix DNA replication‐ associated mutations. This proliferation bias could also enhance the clonal expansion of cells with a proliferative advantage conferred by genetic instability as the surrounding cells grow very slowly. In our study, we cultured cells in platelet lysate‐containing medium and did not observe any difference in proliferation rate between ADSCs cultured under 21% or 1% oxygen (population doubling time ranging from 20 hours at P1 to 40 hours at P10; final cumulative population doublings reaching 33 ± 1 vs. 34 ± 1 at P10). We can therefore directly compare the effect of oxygen concentration on other physiological processes, such as DNA replication or genomic stability, without any proliferation rate‐induced bias.

We also assessed gene expression using RNA‐seq, with the aim to identify potential biomarkers that could account for high oxygen concentration‐induced DNA replication stress and that could be used to assess cancer risk in cellular therapies or tissue regeneration trials before the expanded therapeutic cells are transferred to patients. By comparing gene expression profiles of ADSCs cultured under 21% and 1% oxygen, we established a list of 747 genes that were differentially expressed. As previously reported by Estrada et al., pathway enrichment analysis revealed that genes involved in glycolysis were significantly enriched 9. Furthermore, we identified an enrichment of genes involved in cell cycle control or DNA replication, with most DNA replication genes downregulated in cells grown under the atmospheric oxygen concentration. This is in contrast to the study by Estrada et al., who observed no enrichment of genes in such pathways. However, the authors cultured hMSCs in FBS‐containing medium, which limits cellular proliferation, especially under normoxia. Moreover, they performed microarray‐based expression analysis instead of RNA‐seq. Because the expression of most DNA replication genes is restricted to the S‐phase, an RNA‐seq approach, more sensible than an expression microarray, offers greater potential to reveal differential expression patterns of DNA replication genes between cells cultured under the normoxic and hypoxic conditions. The massive downregulation of DNA replication genes detected is an important observation that concurs with the DNA replication stress observed in ADSCs expanded under the atmospheric oxygen concentration. Among the downregulated genes, we identified two genes coding for components of the replicative helicase complex, *MCM3* and *GINS3*, whose downregulation has been shown to increase DNA replication fork rate, activating the DNA damage response pathways and sensitizing cells to DNA replication inhibitors 43, 44. Moreover, *MCM* downregulation during ageing of hematopoietic stem cells activates the DNA damage response and increases DNA replication fork rate as well as fork stalling 45. Interestingly, we found both increased DNA replication fork rate and fork stalling in ADSCs cultured under an atmospheric oxygen concentration (Fig. 2B and supplemental online Fig. 1). Replicative helicase components downregulated in ADSCs cultured under 21% oxygen concentration may therefore, at least in part, account for the DNA replication stress and DNA damage response pathway activation observed.

To investigate whether high oxygen‐induced DNA replication stress could result in genetic instability in ADSCs, we first performed a CGH array to evaluate genomic stability during cell culture under normoxic and hypoxic conditions. Under neither 1% nor 21% oxygen did we find any significant differences in the genomic profiles at different passages. However, because the in vitro expansion was performed in the absence of any strong selective pressure, we cannot rule out that chromosomal rearrangements, nondetectable with a CGH array resolution, might occur in a subpopulation for which they could offer a proliferation advantage if reimplanted (inoculated in patient) in vivo, where the selective pressure is much higher. Moreover, one of the consequences of replication stress is chromosomal translocation, which is widely observed in cancer cells. It would be interesting to perform exome sequencing to identify and quantify such gene fusions likely to appear in a subset of ADSCs cultured under 21% or 1% oxygen.

We also used the RNA‐seq data to look for SNPs at P4 that were not present at P1 and could therefore be considered as point mutations. We classified these new SNPs into different groups and observed that ADSCs expanded under 21% oxygen accumulated more transversion mutations. Because transversions are more likely to result in amino acid substitutions (as a result of wobble base pair), it is not surprising that more coding mutations were found in ADSCs expanded under 21% oxygen than those cultured under 1% oxygen. Moreover, we found that cells grown under normoxia exhibited more G‐to‐T transversions. Interestingly, it has been shown that oxidative DNA lesions induce predominantly G‐to‐T transversions 46. Our analysis of the point mutation spectrum therefore suggests that oxidative DNA lesions persist when cells are cultured in a 21% oxygen environment and induce point mutations, such as G‐to‐T transversions, even if at a fairly modest level, but significantly at each S‐phase, therefore accumulating during the course of the culture.

## Conclusion

Our study shows that culture of ADSCs under an atmospheric oxygen concentration increases oxidative DNA lesions that could alter DNA replication, activate the DNA damage response, and induce the accumulation of point mutations. Therefore, expanding ADSCs at a low oxygen concentration could reduce the risk for DNA replication stress‐associated transformation, as occurs in neoplastic tissues. Standardized culture methods should be used for hMSC expansion to ensure safe cellular therapies. Here, we suggest using a replicative helicase component gene expression profile and 53BP1 body quantification in G_1_ cells as biomarkers to monitor DNA replication stress associated with ex vivo expansion of hMSCs.

## Author Contributions

R.B.: conception and design, collection and assembly of data, data analysis and interpretation, manuscript writing; M.‐L.R. and L.S.: provision of study material or patients; C.H. and I.G.: data analysis and interpretation; N.J. and M.L.: collection and assembly of data; C.C.: conception and design, manuscript writing; J.‐S.H.: conception and design, manuscript writing, final approval of manuscript.

## Disclosures of Potential Conflicts of Interest

The authors indicated no potential conflicts of interest.

## Supporting information

Supporting Information
