# Supplementary material for: Human Adipose‐Derived Stem Cells Expanded Under Ambient Oxygen Concentration Accumulate Oxidative DNA Lesions and Experience Procarcinogenic DNA Replication Stress
Source: Stem Cells Transl Med. 2016 Aug 2;6(1):68–76. doi: 10.5966/sctm.2015-0401 (PMC5442744; doi:10.5966/sctm.2015-0401)
Supplement: Supplementary file 1 — Supporting Information [file SCT3-6-068-s001.pdf]

Supplementary Figure 1

A

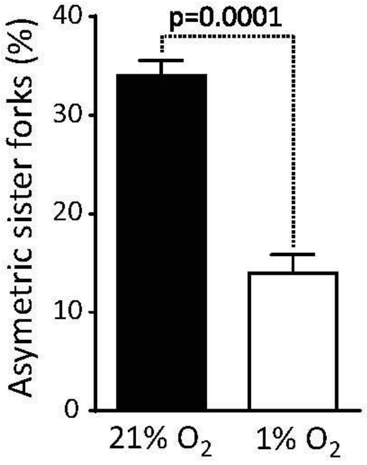

B

	[O2]	Median track length (kb)	Fork velocity (kb/min)	n	p-value
Donor-1	21%	39.05 +/- 1.23	1.953	100	-
	1%	34.29 +/- 0.94	1.715	113	0.0098
Donor-2	21%	43.02 +/- 1.21	2.151	125	-
	1%	35.60 +/- 1.11	1.780	113	< 0.0001
Donor-3	21%	39.30 +/- 1.10	1.965	115	-
	1%	31.42 +/- 0.76	1.571	129	< 0.0001

Fig. S1. Culture of ADSC under 21% oxygen increase both DNA replication fork stalling and speed. **(A)** Proportion of asymmetric sister forks. Error bars represent standard deviation from three independent donors and significance was determined by t-test. **(B)** Replication fork speed in ADSC from three donors cultured under 1% or 21% oxygen. N tracks of IdU and CldU were scored in each condition. The median value of the population is given in kb +/- standard errors and converted in kb/min. Mann-Whitney test was applied to compare data sets, p-value is indicated.

Supplementary Figure 2

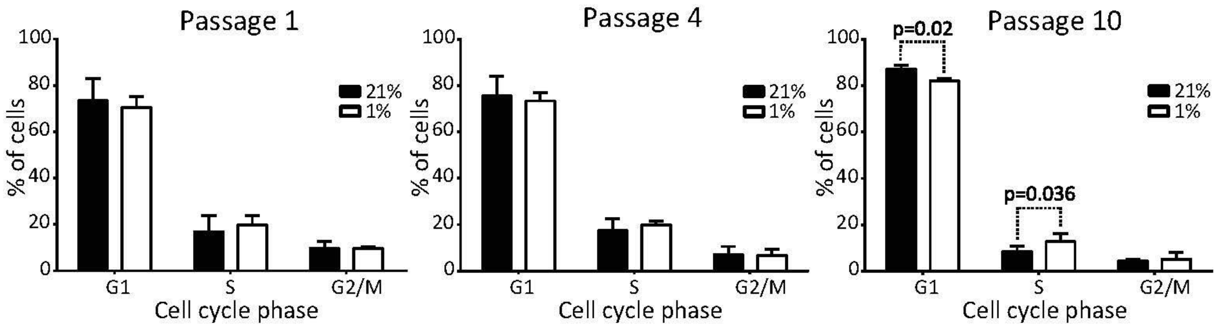

Fig. S2. ADSC grown in Normoxia or hypoxia have similar cell cycle repartition at passage 1 and 4 but not passage 10. Cell cycle repartition of ADSC of three independent donors at passage 1, 4 and 10. Error bars represent standard deviation from the three independent donors and significance was determined by t-test.

Supplementary Figure 3

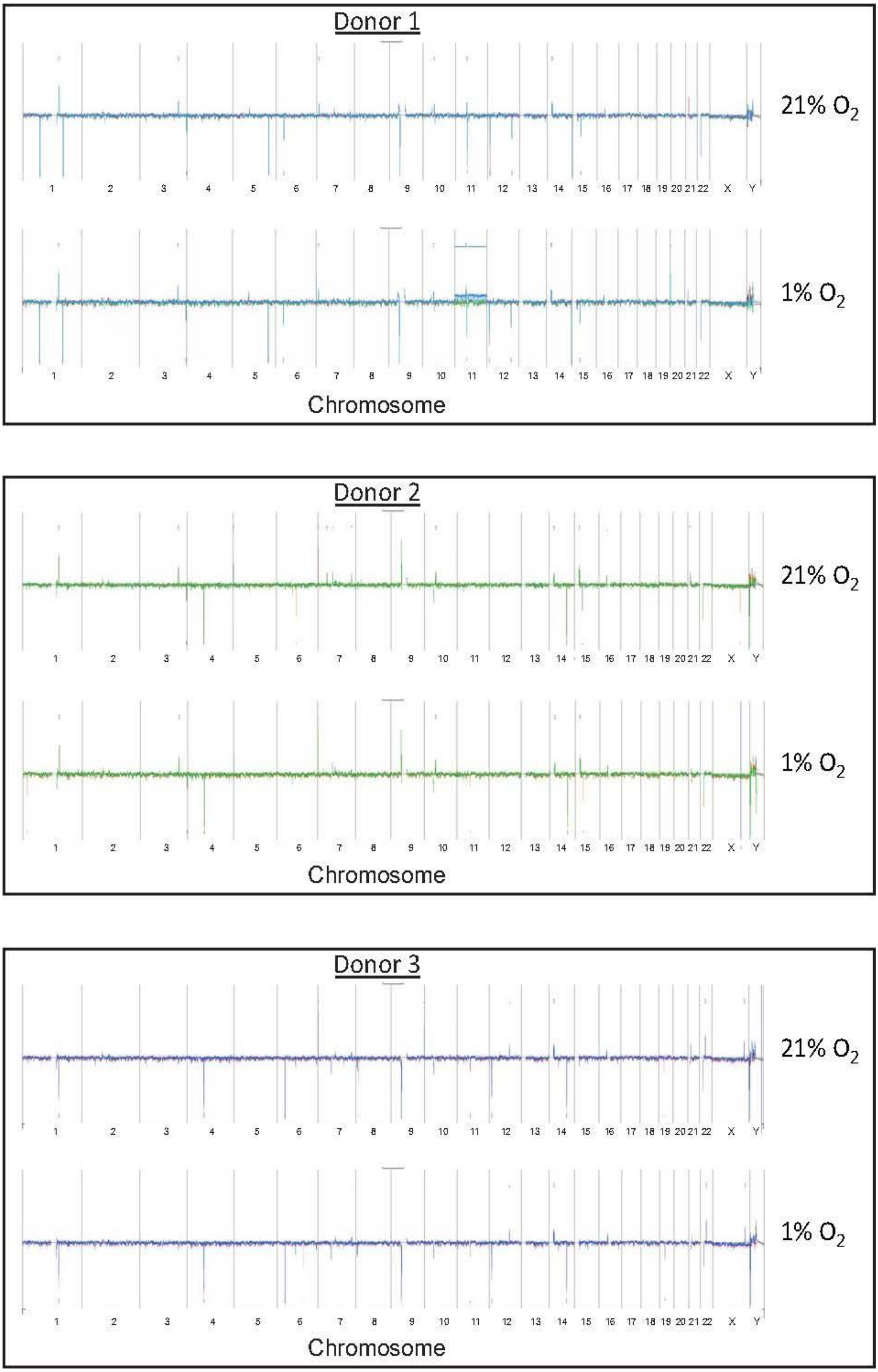

Supplementary Figure 3

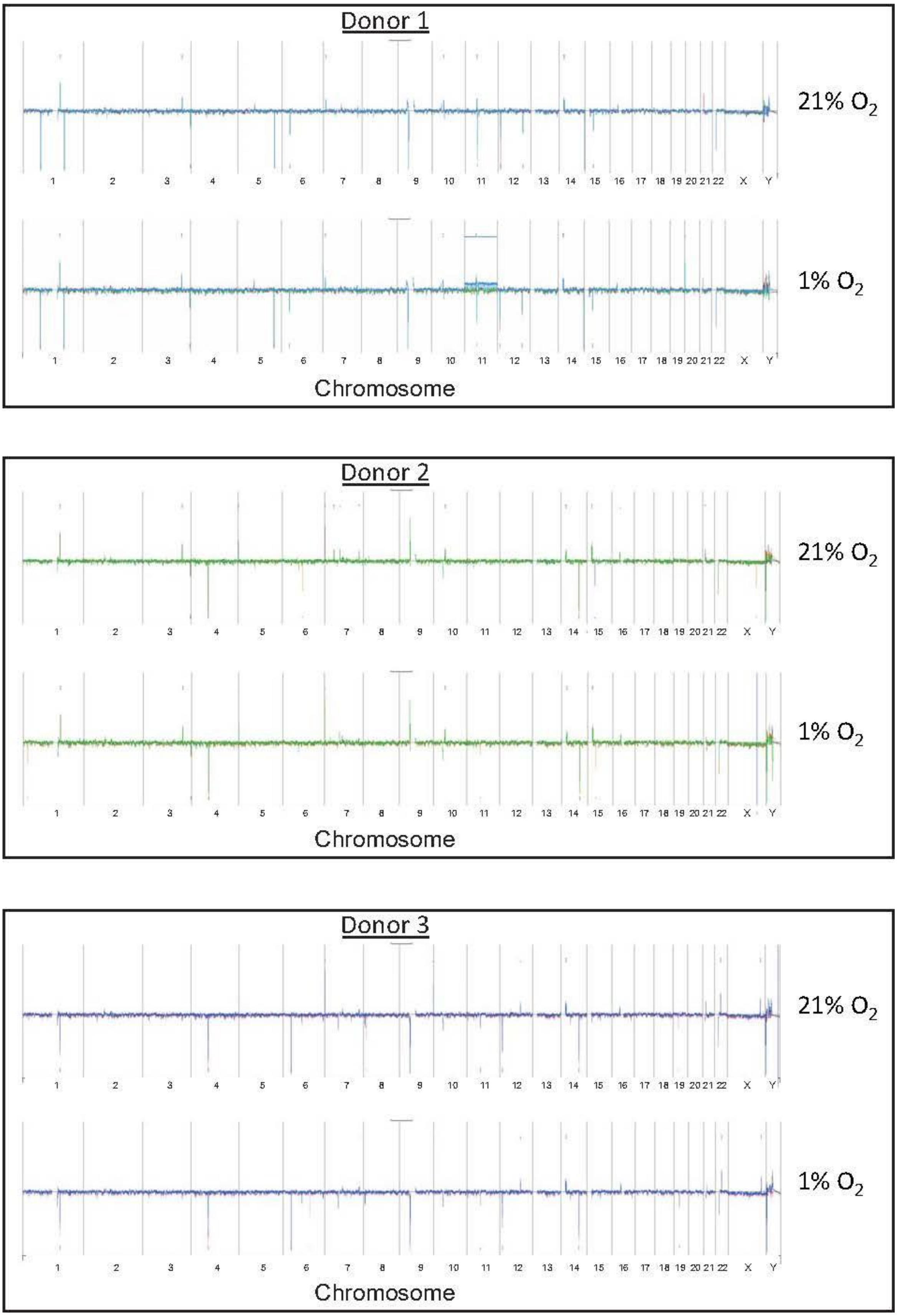

Fig. S3. ADSC do not present significant changes in DNA copy-number variations during long term *ex vivo* expansion. CGH array profiles for donor 1, donor 2 and donor 3. For donor-1, red line corresponds to P1; green line to P4 and blue line to P10. For donor-2, blue line corresponds to P1; orange line to P4 and green line to P10. For donor-3, green line corresponds to P1, pink line to P4 and blue line to P10.

Supplementary Table 1

Gene Symbol	Gene Name	EntrezGene ID	Fold Change	FDR q-value
PPFIA4	protein tyrosine phosphatase, receptor type, f polypeptide (PTPRF), interacting protein (liprin), alpha 4	8497	28.23257866	2.92E-52
PDK1	pyruvate dehydrogenase kinase, isozyme 1	5163	5.540061532	1.80E-27
ZNF395	zinc finger protein 395	55893	4.696747384	8.80E-27
BNIP3P1	BCL2/adenovirus E1B 19kDa interacting protein 3 pseudogene 1	319138	5.102562682	5.18E-19
MIR210HG	MIR210 host gene (non-protein coding)	100506211	9.808197298	1.23E-17
BNIP3	BCL2/adenovirus E1B 19kDa interacting protein 3	664	4.630702263	1.30E-16
EVA1B	eva-1 homolog B (C. elegans)	55194	3.497458648	1.40E-16
VEPH1	ventricular zone expressed PH domain homolog 1 (zebrafish)	79674	0.113236659	2.53E-16
PIK3R6	phosphoinositide-3-kinase, regulatory subunit 6	146850	44.02749935	5.57E-16
CSNK1D	casein kinase 1, delta	1453	6.742828949	6.74E-16
SERPINE2	serpin peptidase inhibitor, clade E (nexin, plasminogen activator inhibitor type 1), member 2	5270	0.103014762	1.03E-14
TAF9B	TAF9B RNA polymerase II, TATA box binding protein (TBP)-associated factor, 31kDa	51616	6.127459315	4.02E-14
SLC2A1-AS1	SLC2A1 antisense RNA 1	440584	3.805753181	5.00E-14
SYNPO	synaptopodin	11346	8.435683691	3.32E-13
PFKFB3	6-phosphofructo-2-kinase/fructose-2,6-biphosphatase 3	5209	4.748335914	5.63E-13
SLC2A1	solute carrier family 2 (facilitated glucose transporter), member 1	6513	3.671709705	2.56E-12
NT5DC1	5'-nucleotidase domain containing 1	221294	7.281718654	2.04E-10

Supplementary Table 1

LURAP1L	leucine rich adaptor protein 1-like	286343	0.100276886	3.31E-09
AK4	adenylate kinase 4	205	3.395113959	4.26E-09
TREM1	triggering receptor expressed on myeloid cells 1	54210	1544.260045	5.52E-09
SLC17A9	solute carrier family 17, member 9	63910	5.460757154	8.56E-09
MT-ND4L	mitochondrially encoded NADH 4L dehydrogenase	4539	704.7901738	8.95E-09
FTH1P2	ferritin, heavy polypeptide 1 pseudogene 2	2497	0.425535088	8.95E-09
BEST1	bestrophin 1	7439	0.425216947	1.55E-08
COL14A1	collagen, type XIV, alpha 1	7373	0.101882135	1.74E-08
ASPN	asporin	54829	0.031522374	1.74E-08
SPSB2	splA/ryanodine receptor domain and SOCS box containing 2	84727	7.501655441	2.33E-08
ANO1-AS1	ANO1 antisense RNA 1	100873981	22.60604962	2.68E-08
KRTAP21-3	keratin associated protein 21-3	100288323	114.6722103	2.70E-08
FBLN7	fibulin 7	129804	5.069094291	2.88E-08
LDHA	lactate dehydrogenase A	3939	2.159240586	4.03E-08
PMEPA1	prostate transmembrane protein, androgen induced 1	56937	3.441408137	6.52E-08
PIK3R5	phosphoinositide-3-kinase, regulatory subunit 5	23533	34.55182477	6.64E-08
TPI1P1	triosephosphate isomerase 1 pseudogene 1	729708	2.218436103	7.48E-08
RUVBL2	RuvB-like 2 (E. coli)	10856	2.166717403	1.12E-07
B4GALNT1	beta-1,4-N-acetyl-galactosaminyl transferase 1	2583	4.674564615	1.87E-07
RASSF2	Ras association (RalGDS/AF-6) domain family member 2	9770	0.150159119	2.22E-07

Supplementary Table 1

GPR1	G protein-coupled receptor 1	2825	0.176736142	3.54E-07
HTRA3	HtrA serine peptidase 3	94031	0.225810112	3.63E-07
CLU	clusterin	1191	0.229976763	4.12E-07
ENPP2	ectonucleotide pyrophosphatase/phosphodiesterase 2	5168	0.268540576	4.59E-07
ADD3	adducin 3 (gamma)	120	0.41706698	6.76E-07
CCL28	chemokine (C-C motif) ligand 28	56477	14.76877233	6.76E-07
FAT4	FAT tumor suppressor homolog 4 (Drosophila)	79633	0.270137906	7.68E-07
ALDOA	aldolase A, fructose-bisphosphate	226	2.045701998	7.94E-07
EGLN1	egl nine homolog 1 (C. elegans)	54583	1.94459619	7.94E-07
VAT1L	vesicle amine transport protein 1 homolog (T. californica)-like	57687	4.06229497	8.30E-07
IL11	interleukin 11	3589	7.605293896	1.03E-06
PTGS2	prostaglandin-endoperoxide synthase 2 (prostaglandin G/H synthase and cyclooxygenase)	5743	6.49733909	1.11E-06
GCSAM	germinal center-associated, signaling and motility	257144	4.342711159	1.11E-06
FTH1P20	ferritin, heavy polypeptide 1 pseudogene 20	729009	0.37386077	1.26E-06
ZC3HAV1L	zinc finger CCCH-type, antiviral 1-like	92092	2.032662934	1.26E-06
APLN	apelin	8862	10.60360552	1.95E-06
TGFBR3	transforming growth factor, beta receptor III	7049	0.266011957	2.11E-06
ACE	angiotensin I converting enzyme (peptidyl-dipeptidase A) 1	1636	0.189316754	2.11E-06
MT-CYB	mitochondrially encoded cytochrome b	4519	8837.361594	2.32E-06

Supplementary Table 1

SECTM1	secreted and transmembrane 1	6398	0.099958219	2.44E-06
TSKU	tsukushi small leucine rich proteoglycan homolog (Xenopus laevis)	25987	2.225573273	2.68E-06
DSP	desmoplakin	1832	5.302909031	2.87E-06
MT-ND4	mitochondrially encoded NADH dehydrogenase 4	4538	10330.8404	2.88E-06
MAP1A	microtubule-associated protein 1A	4130	0.475867225	3.05E-06
ANGPTL1	angiopoietin-like 1	9068	0.205685928	3.05E-06
FHDC1	FH2 domain containing 1	85462	0.149888126	3.12E-06
KIF21B	kinesin family member 21B	23046	11.74091548	3.32E-06
STARD8	StAR-related lipid transfer (START) domain containing 8	9754	5.158769548	3.32E-06
LINC00565	long intergenic non-protein coding RNA 565	100861555	2.859736061	3.32E-06
AUTS2	autism susceptibility candidate 2	26053	2.115683763	3.45E-06
MLPH	melanophilin	79083	27.12832372	4.02E-06
ASAP3	ArfGAP with SH3 domain, ankyrin repeat and PH domain 3	55616	0.313476457	4.38E-06
PTPRF	protein tyrosine phosphatase, receptor type, F	5792	2.906822543	4.42E-06
ALCAM	activated leukocyte cell adhesion molecule	214	2.693798614	4.46E-06
GPX3	glutathione peroxidase 3 (plasma)	2878	1.986616777	5.70E-06
CDCP1	CUB domain containing protein 1	64866	7.952855731	5.95E-06
ECM2	extracellular matrix protein 2, female organ and adipocyte specific	1842	0.129774695	7.18E-06
PPP1R3C	protein phosphatase 1, regulatory subunit 3C	5507	0.255881367	8.72E-06
INHBA-AS1	INHBA antisense RNA 1	285954	4.711629183	1.05E-05

Supplementary Table 1

HS3ST3B1	heparan sulfate (glucosamine) 3-O-sulfotransferase 3B1	9953	2.211866021	1.05E-05
NRIP3	nuclear receptor interacting protein 3	56675	2.882073244	1.10E-05
NOTCH3	notch 3	4854	5.513834634	1.37E-05
LMO4	LIM domain only 4	8543	2.171706289	1.38E-05
SLC8A1	solute carrier family 8 (sodium/calcium exchanger), member 1	6546	30.74114508	1.57E-05
PLA2G16	phospholipase A2, group XVI	11145	0.223768831	1.57E-05
MPP2	membrane protein, palmitoylated 2 (MAGUK p55 subfamily member 2)	4355	2.89631597	1.86E-05
MYEOV	myeloma overexpressed (in a subset of t(11;14) positive multiple myelomas)	26579	5.960139095	2.05E-05
THUMPD2	THUMP domain containing 2	80745	2.141421809	2.19E-05
ENO1	enolase 1, (alpha)	2023	2.067639975	2.33E-05
SLC38A1	solute carrier family 38, member 1	81539	0.481644301	2.33E-05
DTX3	deltex homolog 3 (Drosophila)	196403	1.920389696	2.43E-05
VIL1	villin 1	7429	1.925381989	2.86E-05
PSG5	pregnancy specific beta-1-glycoprotein 5	5673	10.90575692	3.04E-05
ITGA11	integrin, alpha 11	22801	0.319333099	3.04E-05
BCOR	BCL6 corepressor	54880	1.882441459	3.05E-05
SAMD9L	sterile alpha motif domain containing 9-like	219285	0.344555191	3.11E-05
CTSC	cathepsin C	1075	0.455845235	3.22E-05
INSIG2	insulin induced gene 2	51141	2.071610822	3.38E-05
TLR3	toll-like receptor 3	7098	0.206713999	3.40E-05

Supplementary Table 1

CTH	cystathionase (cystathionine gamma-lyase)	1491	0.354087053	4.93E-05
IGFBP4	insulin-like growth factor binding protein 4	3487	0.486496051	5.13E-05
PITRM1	pitrilysin metallopeptidase 1	10531	2.19577465	5.98E-05
C21orf2	chromosome 21 open reading frame 2	755	2.100354721	6.03E-05
EFEMP1	EGF containing fibulin-like extracellular matrix protein 1	2202	0.202739485	6.03E-05
STC1	stanniocalcin 1	6781	21.73947206	6.07E-05
SNAPC5	small nuclear RNA activating complex, polypeptide 5, 19kDa	10302	1.936160297	6.49E-05
RAB30	RAB30, member RAS oncogene family	27314	0.366407007	7.51E-05
LGR4	leucine-rich repeat containing G protein-coupled receptor 4	55366	0.210158105	7.63E-05
IGFBPL1	insulin-like growth factor binding protein-like 1	347252	10.16570947	7.67E-05
HS3ST3A1	heparan sulfate (glucosamine) 3-O-sulfotransferase 3A1	9955	2.492763476	7.67E-05
GPI	glucose-6-phosphate isomerase	2821	1.910098583	7.67E-05
RMDN2	regulator of microtubule dynamics 2	151393	0.099019673	7.78E-05
DAB1	disabled homolog 1 (Drosophila)	1600	0.447382894	8.66E-05
WNT7B	wingless-type MMTV integration site family, member 7B	7477	6.430718777	1.02E-04
RPL21P23	ribosomal protein L21 pseudogene 23	100270879	0.330347653	1.06E-04
CNKSR3	CNKSR family member 3	154043	0.301992578	1.17E-04
CMBL	carboxymethylenebutenolidase homolog (Pseudomonas)	134147	0.480268802	1.17E-04
ORC4	origin recognition complex, subunit 4	5000	0.266621865	1.17E-04
GDNF	glial cell derived neurotrophic factor	2668	5.076288275	1.19E-04

Supplementary Table 1

RTN4IP1	reticulon 4 interacting protein 1	84816	0.446185953	1.44E-04
DCLK1	doublecortin-like kinase 1	9201	0.224665266	1.53E-04
ZNF710	zinc finger protein 710	374655	2.199829941	1.79E-04
DNAH11	dynein, axonemal, heavy chain 11	8701	1.771093896	1.83E-04
MBNL1	muscleblind-like splicing regulator 1	4154	0.433974747	1.83E-04
PI16	peptidase inhibitor 16	221476	0.074207149	1.83E-04
C4orf3	chromosome 4 open reading frame 3	401152	1.832802841	1.85E-04
CCDC152	coiled-coil domain containing 152	100129792	0.071574773	2.02E-04
TRAK1	trafficking protein, kinesin binding 1	22906	0.519676959	2.03E-04
PGM1	phosphoglucomutase 1	5236	1.822381365	2.08E-04
ZNF229	zinc finger protein 229	7772	0.236550317	2.31E-04
PARP14	poly (ADP-ribose) polymerase family, member 14	54625	0.506878825	2.41E-04
CDKN2B	cyclin-dependent kinase inhibitor 2B (p15, inhibits CDK4)	1030	0.231154123	2.46E-04
IL20RB-AS1	IL20RB antisense RNA 1	100862678	0.375142339	2.75E-04
OMD	osteomodulin	4958	0.02237172	2.76E-04
SQRDL	sulfide quinone reductase-like (yeast)	58472	0.380208318	2.81E-04
EPAS1	endothelial PAS domain protein 1	2034	0.416560515	3.02E-04
BEND5	BEN domain containing 5	79656	11.57787034	3.05E-04
P4HA2-AS1	P4HA2 antisense RNA 1	100861518	1.954531045	3.05E-04
LDHAP7	lactate dehydrogenase A pseudogene 7	100190800	2.144630342	3.05E-04

Supplementary Table 1

CACNB4	calcium channel, voltage-dependent, beta 4 subunit	785	0.153734727	3.05E-04
MCU	mitochondrial calcium uniporter	90550	0.513189337	3.14E-04
PTK7	PTK7 protein tyrosine kinase 7	5754	2.123836931	3.23E-04
SPA17	sperm autoantigenic protein 17	53340	0.548349531	3.26E-04
CREG1	cellular repressor of E1A-stimulated genes 1	8804	0.448244877	3.43E-04
ME1	malic enzyme 1, NADP(+)-dependent, cytosolic	4199	0.481627942	3.47E-04
KCNA3	potassium voltage-gated channel, shaker-related subfamily, member 3	3738	4.113897931	3.71E-04
UBE2L6	ubiquitin-conjugating enzyme E2L 6	9246	0.489129031	4.18E-04
TNFRSF10D	tumor necrosis factor receptor superfamily, member 10d, decoy with truncated death domain	8793	3.35735013	4.31E-04
RHOJ	ras homolog family member J	57381	0.418840251	4.78E-04
RNF145	ring finger protein 145	153830	1.650282031	5.13E-04
IFI35	interferon-induced protein 35	3430	0.584295993	5.17E-04
ISCU	iron-sulfur cluster scaffold homolog (E. coli)	23479	0.604760744	5.28E-04
GABRE	gamma-aminobutyric acid (GABA) A receptor, epsilon	2564	0.35470658	5.28E-04
ST3GAL5	ST3 beta-galactoside alpha-2,3-sialyltransferase 5	8869	0.349371783	5.48E-04
VAT1	vesicle amine transport protein 1 homolog (T. californica)	10493	0.586573703	5.64E-04
TMEM63A	transmembrane protein 63A	9725	0.457711359	5.74E-04
DUSP4	dual specificity phosphatase 4	1846	6.382873253	5.79E-04
AIF1L	allograft inflammatory factor 1-like	83543	0.046994769	5.98E-04

Supplementary Table 1

PGAM1P5	phosphoglycerate mutase 1 pseudogene 5	100132594	0.1332846	6.06E-04
FAM198B	family with sequence similarity 198, member B	51313	0.541146605	6.09E-04
SLC5A10	solute carrier family 5 (sodium/glucose cotransporter), member 10	125206	2.073613477	6.56E-04
RNF215	ring finger protein 215	200312	1.928032272	6.56E-04
C1orf170	chromosome 1 open reading frame 170	84808	3.2906441	6.82E-04
CASP1	caspase 1, apoptosis-related cysteine peptidase	834	0.307234259	6.82E-04
NCOA7	nuclear receptor coactivator 7	135112	0.529435265	6.85E-04
TLDC2	TBC/LysM-associated domain containing 2	140711	0.560475727	7.00E-04
MIR3124	microRNA 3124	100422879	2.168845072	7.14E-04
PFKL	phosphofructokinase, liver	5211	2.015603686	7.30E-04
EXTL1	exostoses (multiple)-like 1	2134	4.847302104	7.72E-04
LZTS1	leucine zipper, putative tumor suppressor 1	11178	2.981493104	7.72E-04
AHNAK2	AHNAK nucleoprotein 2	113146	0.428125597	8.16E-04
MMS22L	MMS22-like, DNA repair protein	253714	2.019865179	8.57E-04
GSTA4	glutathione S-transferase alpha 4	2941	0.608983608	8.62E-04
SSTR1	somatostatin receptor 1	6751	7.736741117	8.82E-04
TPGS2	tubulin polyglutamylase complex subunit 2	25941	1.970267069	8.95E-04
CAMKK1	calcium/calmodulin-dependent protein kinase kinase 1, alpha	84254	3.135370645	9.72E-04
SLC7A14	solute carrier family 7 (orphan transporter), member 14	57709	0.374362025	9.82E-04
MGST3	microsomal glutathione S-transferase 3	4259	0.627973245	1.06E-03

Supplementary Table 1

NTF3	neurotrophin 3	4908	3.342614546	1.08E-03
SAMD9	sterile alpha motif domain containing 9	54809	0.452431097	1.11E-03
DNAJC19P1	DnaJ (Hsp40) homolog, subfamily C, member 19 pseudogene 1	100129853	0.242663159	1.11E-03
KDM3A	lysine (K)-specific demethylase 3A	55818	1.672504277	1.14E-03
SHROOM2	shroom family member 2	357	9.846738876	1.17E-03
CYP21A2	cytochrome P450, family 21, subfamily A, polypeptide 2	1589	0.020067518	1.18E-03
SGK223	homolog of rat pragma of Rnd2	157285	2.252114223	1.23E-03
PEG10	paternally expressed 10	23089	0.479818936	1.23E-03
OSR2	odd-skipped related 2 (Drosophila)	116039	0.269938376	1.23E-03
SEMA7A	semaphorin 7A, GPI membrane anchor (John Milton Hagen blood group)	8482	4.148066434	1.25E-03
APCDD1L	adenomatosis polyposis coli down-regulated 1-like	164284	3.157997436	1.25E-03
MYBL1	v-myb myeloblastosis viral oncogene homolog (avian)-like 1	4603	2.55220462	1.27E-03
MT-ND5	mitochondrially encoded NADH dehydrogenase 5	4540	7.057831562	1.38E-03
PDE4D	phosphodiesterase 4D, cAMP-specific	5144	2.847654268	1.47E-03
WDR63	WD repeat domain 63	126820	0.247680123	1.57E-03
PGD	phosphogluconate dehydrogenase	5226	0.53034604	1.63E-03
SCARA5	scavenger receptor class A, member 5 (putative)	286133	0.010000383	1.71E-03
CMTM4	CKLF-like MARVEL transmembrane domain containing 4	146223	1.645893629	1.72E-03
DCK	deoxycytidine kinase	1633	2.053433177	1.75E-03
C19orf60	chromosome 19 open reading frame 60	55049	2.585590854	1.78E-03

Supplementary Table 1

NRBF2	nuclear receptor binding factor 2	29982	0.59468995	1.85E-03
PAPD7	PAP associated domain containing 7	11044	1.610440322	1.85E-03
HAS2	hyaluronan synthase 2	3037	3.318892728	1.89E-03
IDH1	isocitrate dehydrogenase 1 (NADP+), soluble	3417	0.578957038	1.93E-03
IFFO1	intermediate filament family orphan 1	25900	1.618620867	1.95E-03
CLDN11	claudin 11	5010	0.382656815	1.95E-03
GULP1	GULP, engulfment adaptor PTB domain containing 1	51454	0.297824016	1.95E-03
XYLT2	xylosyltransferase II	64132	1.996464612	1.96E-03
SIPA1L1	signal-induced proliferation-associated 1 like 1	26037	1.867799375	1.96E-03
MGST1	microsomal glutathione S-transferase 1	4257	0.593888046	1.96E-03
PTGIS	prostaglandin I2 (prostacyclin) synthase	5740	0.200281768	1.96E-03
PKMYT1	protein kinase, membrane associated tyrosine/threonine 1	9088	2.868580491	2.04E-03
S100A10	S100 calcium binding protein A10	6281	0.500172935	2.04E-03
HAS2-AS1	HAS2 antisense RNA 1	594842	3.283742499	2.05E-03
FLRT2	fibronectin leucine rich transmembrane protein 2	23768	0.464074715	2.05E-03
SPATA6L	spermatogenesis associated 6-like	55064	2.166056688	2.14E-03
FAM46A	family with sequence similarity 46, member A	55603	0.529077841	2.14E-03
ADAM19	ADAM metallopeptidase domain 19	8728	2.591517256	2.19E-03
RS1	retinoschisin 1	6247	1.742486526	2.20E-03
LRRC8D	leucine rich repeat containing 8 family, member D	55144	0.554126128	2.24E-03

Supplementary Table 1

SLC4A4	solute carrier family 4, sodium bicarbonate cotransporter, member 4	8671	1.869107828	2.41E-03
PTGFR	prostaglandin F receptor (FP)	5737	0.265849194	2.43E-03
CXADR	coxsackie virus and adenovirus receptor	1525	1.731605994	2.43E-03
FAM129B	family with sequence similarity 129, member B	64855	1.930783362	2.45E-03
COL27A1	collagen, type XXVII, alpha 1	85301	2.056783583	2.50E-03
COPA	coatomer protein complex, subunit alpha	1314	0.654285317	2.50E-03
COL7A1	collagen, type VII, alpha 1	1294	2.497883168	2.55E-03
LRRN4CL	LRRN4 C-terminal like	221091	0.152060569	2.55E-03
SDC4	syndecan 4	6385	1.778258796	2.55E-03
BRIP1	BRCA1 interacting protein C-terminal helicase 1	83990	2.042866602	2.63E-03
DUTP2	deoxyuridine triphosphatase pseudogene 2	100873909	1.972697575	2.64E-03
CCDC158	coiled-coil domain containing 158	339965	2.20137969	2.65E-03
KIAA1715	KIAA1715	80856	1.61537207	2.66E-03
LINC00702	long intergenic non-protein coding RNA 702	100652988	2.053882999	2.73E-03
FAM129A	family with sequence similarity 129, member A	116496	0.524079107	2.74E-03
SERPINB2	serpin peptidase inhibitor, clade B (ovalbumin), member 2	5055	17.19813238	2.76E-03
PLOD1	procollagen-lysine, 2-oxoglutarate 5-dioxygenase 1	5351	1.787208683	2.93E-03
XPNPEP2	X-prolyl aminopeptidase (aminopeptidase P) 2, membrane-bound	7512	0.196248972	2.94E-03
MR1	major histocompatibility complex, class I-related	3140	0.548984377	2.97E-03
ANXA4	annexin A4	307	0.617769478	2.99E-03

Supplementary Table 1

GRHL1	grainyhead-like 1 (Drosophila)	29841	0.222229462	2.99E-03
PAPPA-AS1	PAPPA antisense RNA 1	493913	0.398197757	3.00E-03
ZNF224	zinc finger protein 224	7767	0.258137206	3.00E-03
H6PD	hexose-6-phosphate dehydrogenase (glucose 1-dehydrogenase)	9563	1.712412177	3.11E-03
E2F7	E2F transcription factor 7	144455	2.776098966	3.12E-03
FOXC1	forkhead box C1	2296	0.412474833	3.13E-03
HILPDA	hypoxia inducible lipid droplet-associated	29923	1.900653178	3.15E-03
RGS16	regulator of G-protein signaling 16	6004	5.843906112	3.15E-03
ORAOV1	oral cancer overexpressed 1	220064	2.6334444	3.15E-03
LDHAP4	lactate dehydrogenase A pseudogene 4	158222	2.132855164	3.18E-03
ATP6V0A1	ATPase, H+ transporting, lysosomal V0 subunit a1	535	0.620469414	3.18E-03
HSPA2	heat shock 70kDa protein 2	3306	3.256061614	3.18E-03
SMAD3	SMAD family member 3	4088	0.487288811	3.21E-03
SLC7A11-AS1	SLC7A11 antisense RNA 1	641364	0.444283356	3.25E-03
PDE1A	phosphodiesterase 1A, calmodulin-dependent	5136	0.225396185	3.27E-03
TNIK	TRAF2 and NCK interacting kinase	23043	0.363927506	3.30E-03
NET1	neuroepithelial cell transforming 1	10276	2.178302025	3.35E-03
ERG	v-ets erythroblastosis virus E26 oncogene homolog (avian)	2078	7.346138011	3.46E-03
EFNB2	ephrin-B2	1948	4.852543009	3.46E-03
ASAH1	N-acylsphingosine amidohydrolase (acid ceramidase) 1	427	0.617920268	3.51E-03

Supplementary Table 1

CAMK1D	calcium/calmodulin-dependent protein kinase ID	57118	0.291632821	3.53E-03
STAT1	signal transducer and activator of transcription 1, 91kDa	6772	0.283761743	3.56E-03
CADPS	Ca++-dependent secretion activator	8618	8.144787168	3.61E-03
MMP14	matrix metallopeptidase 14 (membrane-inserted)	4323	2.134809002	3.69E-03
NUPL1	nucleoporin like 1	9818	1.709972353	3.77E-03
HEATR1	HEAT repeat containing 1	55127	1.568819421	3.77E-03
ZNF608	zinc finger protein 608	57507	0.226552136	3.77E-03
SORBS1	sorbin and SH3 domain containing 1	10580	2.54499355	3.87E-03
CENPJ	centromere protein J	55835	1.853028666	3.87E-03
MRPL51	mitochondrial ribosomal protein L51	51258	1.56701273	3.92E-03
GLT8D2	glycosyltransferase 8 domain containing 2	83468	0.557496634	3.92E-03
IL1RN	interleukin 1 receptor antagonist	3557	15.81400631	4.00E-03
PIGB	phosphatidylinositol glycan anchor biosynthesis, class B	9488	0.532020816	4.05E-03
MTR	5-methyltetrahydrofolate-homocysteine methyltransferase	4548	1.531242524	4.19E-03
A2M	alpha-2-macroglobulin	2	3.748748397	4.35E-03
SLC8A1-AS1	SLC8A1 antisense RNA 1	100128590	2.80107381	4.38E-03
MZT1	mitotic spindle organizing protein 1	440145	1.691951132	4.39E-03
FOXP4	forkhead box P4	116113	1.791038811	4.51E-03
WFDC13	WAP four-disulfide core domain 13	164237	4.601314283	4.61E-03
ARAP3	ArfGAP with RhoGAP domain, ankyrin repeat and PH domain 3	64411	2.04098418	4.61E-03

Supplementary Table 1

TMEM194A	transmembrane protein 194A	23306	1.863794243	4.61E-03
GJC1	gap junction protein, gamma 1, 45kDa	10052	1.859288054	4.61E-03
NT5E	5'-nucleotidase, ecto (CD73)	4907	1.783988728	4.61E-03
ZNF292	zinc finger protein 292	23036	1.599694706	4.61E-03
TMEM119	transmembrane protein 119	338773	0.415571321	4.61E-03
ABHD4	abhydrolase domain containing 4	63874	0.353410302	4.61E-03
CD274	CD274 molecule	29126	5.693634962	4.62E-03
GRIA3	glutamate receptor, ionotropic, AMPA 3	2892	2.458112594	4.73E-03
ASS1	argininosuccinate synthase 1	445	0.365749805	4.83E-03
MGLL	monoglyceride lipase	11343	1.690868307	4.86E-03
FAM115C	family with sequence similarity 115, member C	285966	4.30555435	4.88E-03
DIAPH3-AS1	DIAPH3 antisense RNA 1	100874195	1.789603893	4.88E-03
FBLN1	fibulin 1	2192	0.485153438	4.88E-03
ITPRIPL1	inositol 1,4,5-trisphosphate receptor interacting protein-like 1	150771	3.467430582	4.94E-03
CSF1	colony stimulating factor 1 (macrophage)	1435	0.558258803	4.94E-03
C11orf89	chromosome 11 open reading frame 89	728008	0.233425164	4.94E-03
POLD3	polymerase (DNA-directed), delta 3, accessory subunit	10714	1.739798348	4.97E-03
CFH	complement factor H	3075	0.328522252	5.08E-03
CHAC1	ChaC, cation transport regulator homolog 1 (E. coli)	79094	0.330722705	5.23E-03
HK2P1	hexokinase 2 pseudogene 1	642546	4.055157643	5.32E-03

Supplementary Table 1

C2CD2	C2 calcium-dependent domain containing 2	25966	2.240143264	5.32E-03
ARSG	arylsulfatase G	22901	0.623404425	5.32E-03
AP1G2	adaptor-related protein complex 1, gamma 2 subunit	8906	0.547130733	5.32E-03
ITGA3	integrin, alpha 3 (antigen CD49C, alpha 3 subunit of VLA-3 receptor)	3675	3.713969583	5.35E-03
WDR62	WD repeat domain 62	284403	2.370957825	5.43E-03
PAPPA2	pappalysin 2	60676	0.367793528	5.48E-03
LRRC16A	leucine rich repeat containing 16A	55604	0.513824683	5.57E-03
TTC39B	tetratricopeptide repeat domain 39B	158219	0.56457995	5.61E-03
LEPREL1	leprecan-like 1	55214	2.521927846	5.61E-03
LRRC3	leucine rich repeat containing 3	81543	3.763985316	5.62E-03
PPP1R3G	protein phosphatase 1, regulatory subunit 3G	648791	2.65660917	5.85E-03
CLEC14A	C-type lectin domain family 14, member A	161198	7.477956091	5.85E-03
ZNF672	zinc finger protein 672	79894	2.122444751	5.85E-03
PSAT1	phosphoserine aminotransferase 1	29968	0.551443621	5.89E-03
AKR1C2	aldo-keto reductase family 1, member C2 (dihydrodiol dehydrogenase 2; bile acid binding protein; 3-alpha hydroxysteroid dehydrogenase, type III)	1646	0.397409427	6.06E-03
RGL1	ral guanine nucleotide dissociation stimulator-like 1	23179	0.531252194	6.15E-03
RAB3B	RAB3B, member RAS oncogene family	5865	1.808003788	6.25E-03
ANK2	ankyrin 2, neuronal	287	0.536870411	6.38E-03
SYNPO2	synaptopodin 2	171024	0.249083783	6.50E-03

Supplementary Table 1

APBA2	amyloid beta (A4) precursor protein-binding, family A, member 2	321	3.024823578	6.65E-03
TRANK1	tetratricopeptide repeat and ankyrin repeat containing 1	9881	0.468374033	6.65E-03
LSP1	lymphocyte-specific protein 1	4046	0.07034287	6.84E-03
CACNA1A	calcium channel, voltage-dependent, P/Q type, alpha 1A subunit	773	3.173520144	7.24E-03
UHRF1	ubiquitin-like with PHD and ring finger domains 1	29128	2.096251767	7.49E-03
TMEM45A	transmembrane protein 45A	55076	1.827061377	7.49E-03
FAM127C	family with sequence similarity 127, member C	441518	1.683380138	7.63E-03
SESN2	sestrin 2	83667	0.507403262	7.63E-03
PPAPDC1A	phosphatidic acid phosphatase type 2 domain containing 1A	196051	4.797018489	7.65E-03
MYBL2	v-myb myeloblastosis viral oncogene homolog (avian)-like 2	4605	2.04005776	7.65E-03
XAF1	XIAP associated factor 1	54739	0.550604879	7.65E-03
HNMT	histamine N-methyltransferase	3176	0.394283434	7.65E-03
RLBP1	retinaldehyde binding protein 1	6017	1.940476821	7.67E-03
RCAN1	regulator of calcineurin 1	1827	1.856607691	7.67E-03
C9orf24	chromosome 9 open reading frame 24	84688	1.606621625	7.67E-03
COL18A1	collagen, type XVIII, alpha 1	80781	2.170845421	7.73E-03
INCENP	inner centromere protein antigens 135/155kDa	3619	1.819693537	7.83E-03
GLRA2	glycine receptor, alpha 2	2742	2.773325591	7.83E-03
CASP8AP2	caspase 8 associated protein 2	9994	1.556773762	7.83E-03
TRIM5	tripartite motif containing 5	85363	0.611095431	7.87E-03

Supplementary Table 1

EDNRA	endothelin receptor type A	1909	3.787621058	7.91E-03
MIR4524B	microRNA 4524b	100847008	0.208279824	7.94E-03
NCAPG2	non-SMC condensin II complex, subunit G2	54892	1.866004933	7.99E-03
PLK2	polo-like kinase 2	10769	0.515377622	7.99E-03
GAMT	guanidinoacetate N-methyltransferase	2593	0.397332029	8.00E-03
VCAN-AS1	VCAN antisense RNA 1	100873929	0.622814613	8.15E-03
SMAD9	SMAD family member 9	4093	2.385287226	8.17E-03
CLCN2	chloride channel, voltage-sensitive 2	1181	1.774373716	8.26E-03
HEG1	HEG homolog 1 (zebrafish)	57493	0.545941207	8.26E-03
RGMB-AS1	RGMB antisense RNA 1	503569	2.10757212	8.31E-03
BMPER	BMP binding endothelial regulator	168667	0.610655703	8.51E-03
CA12	carbonic anhydrase XII	771	3.915210838	8.61E-03
FMN2	formin 2	56776	3.260635107	8.61E-03
RRM2	ribonucleotide reductase M2	6241	2.311574724	8.76E-03
GALNT15	polypeptide N-acetylgalactosaminyltransferase 15	117248	2.410728197	8.76E-03
RASA3	RAS p21 protein activator 3	22821	1.482903071	8.89E-03
BCAP29	B-cell receptor-associated protein 29	55973	1.541673436	8.91E-03
AMT	aminomethyltransferase	275	0.609523183	8.91E-03
ACY1	aminoacylase 1	95	0.630591094	8.96E-03
TRIM22	tripartite motif containing 22	10346	0.525178223	8.96E-03

Supplementary Table 1

PRELID2	PRELI domain containing 2	153768	2.129484162	9.10E-03
SPRY4	sprouty homolog 4 (Drosophila)	81848	3.032767707	9.31E-03
ARHGEF39	Rho guanine nucleotide exchange factor (GEF) 39	84904	2.351882525	9.31E-03
ARSJ	arylsulfatase family, member J	79642	1.658989091	9.45E-03
SLC25A36	solute carrier family 25 (pyrimidine nucleotide carrier), member 36	55186	1.465610049	9.56E-03
GPX4	glutathione peroxidase 4	2879	0.606425532	9.56E-03
HSPA8P16	heat shock 70kDa protein 8 pseudogene 16	100289643	0.115560479	9.64E-03
POLE	polymerase (DNA directed), epsilon, catalytic subunit	5426	1.840325169	9.71E-03
ADM2	adrenomedullin 2	79924	0.392178676	9.95E-03
PPARG	peroxisome proliferator-activated receptor gamma	5468	0.347523634	9.95E-03
NTM	neurotrimin	50863	2.377837293	1.00E-02
APOL6	apolipoprotein L, 6	80830	0.491215163	1.00E-02
ADH1B	alcohol dehydrogenase 1B (class I), beta polypeptide	125	0.051036351	1.01E-02
PDCD1LG2	programmed cell death 1 ligand 2	80380	3.627958618	1.02E-02
MFAP5	microfibrillar associated protein 5	8076	0.420190369	1.02E-02
VPS53	vacuolar protein sorting 53 homolog (S. cerevisiae)	55275	3.950597864	1.03E-02
MT-ND1	mitochondrially encoded NADH dehydrogenase 1	4535	0.436133888	1.04E-02
ID3	inhibitor of DNA binding 3, dominant negative helix-loop-helix protein	3399	2.844557019	1.05E-02
CHP1	calcineurin-like EF hand protein 1	11261	0.668871417	1.05E-02
ADAMTS15	ADAM metallopeptidase with thrombospondin type 1 motif, 15	170689	0.172386894	1.05E-02

Supplementary Table 1

CYP1B1-AS1	CYP1B1 antisense RNA 1	285154	0.093158227	1.05E-02
RAB11B-AS1	RAB11B antisense RNA 1	100507567	2.809775764	1.05E-02
PITPNM3	PITPNM family member 3	83394	2.245466191	1.05E-02
NBPF2P	neuroblastoma breakpoint family, member 2, pseudogene	343381	0.53101941	1.06E-02
METRNL	meteorin, glial cell differentiation regulator-like	284207	2.25027762	1.07E-02
RNU7-75P	RNA, U7 small nuclear 75 pseudogene	100151672	1.659401965	1.08E-02
UGDH	UDP-glucose 6-dehydrogenase	7358	0.6577146	1.08E-02
ENOX1	ecto-NOX disulfide-thiol exchanger 1	55068	0.511593096	1.08E-02
PTCHD3P1	patched domain containing 3 pseudogene 1	387647	0.256363895	1.09E-02
SEPT5	septin 5	5413	1.92690347	1.10E-02
MCTP1	multiple C2 domains, transmembrane 1	79772	1.732201785	1.13E-02
APOL3	apolipoprotein L, 3	80833	0.290403551	1.16E-02
CCNH	cyclin H	902	1.599185504	1.19E-02
BCL7A	B-cell CLL/lymphoma 7A	605	1.551008508	1.20E-02
MESDC2	mesoderm development candidate 2	23184	0.474456919	1.21E-02
PDGFRA	platelet-derived growth factor receptor, alpha polypeptide	5156	0.421325167	1.21E-02
SLC43A2	solute carrier family 43, member 2	124935	0.317814092	1.22E-02
DAPK1	death-associated protein kinase 1	1612	0.216355695	1.22E-02
RAD51-AS1	RAD51 antisense RNA 1 (head to head)	100505648	1.914021055	1.23E-02
SAP30	Sin3A-associated protein, 30kDa	8819	0.377219981	1.23E-02

Supplementary Table 1

NDRG1	N-myc downstream regulated 1	10397	1.650946649	1.25E-02
ELFN1	extracellular leucine-rich repeat and fibronectin type III domain containing 1	392617	0.196489347	1.28E-02
ALK	anaplastic lymphoma receptor tyrosine kinase	238	2.86221094	1.31E-02
TBX3	T-box 3	6926	1.873069589	1.34E-02
C9orf139	chromosome 9 open reading frame 139	401563	1.659507558	1.35E-02
TRIM69	tripartite motif containing 69	140691	0.596793613	1.35E-02
NCKAP5	NCK-associated protein 5	344148	0.237637101	1.36E-02
ZNF295-AS1	ZNF295 antisense RNA 1	150142	1.713431362	1.36E-02
UAP1L1	UDP-N-acteylglucosamine pyrophosphorylase 1-like 1	91373	1.896463362	1.38E-02
MEF2D	myocyte enhancer factor 2D	4209	1.502827686	1.38E-02
INHBA	inhibin, beta A	3624	3.721187279	1.39E-02
PLAUR	plasminogen activator, urokinase receptor	5329	1.88038138	1.39E-02
C14orf79	chromosome 14 open reading frame 79	122616	1.868341528	1.41E-02
IL17RD	interleukin 17 receptor D	54756	0.484478987	1.42E-02
ADH5	alcohol dehydrogenase 5 (class III), chi polypeptide	128	0.602292542	1.42E-02
AGPAT5	1-acylglycerol-3-phosphate O-acyltransferase 5 (lysophosphatidic acid acyltransferase, epsilon)	55326	1.87157894	1.44E-02
TMEM201	transmembrane protein 201	199953	1.919128215	1.48E-02
ZNF773	zinc finger protein 773	374928	0.509908891	1.49E-02
CENPI	centromere protein I	2491	1.986854464	1.50E-02

Supplementary Table 1

COL25A1	collagen, type XXV, alpha 1	84570	4.81259827	1.52E-02
TNFAIP6	tumor necrosis factor, alpha-induced protein 6	7130	0.642596666	1.52E-02
LBH	limb bud and heart development homolog (mouse)	81606	2.298792106	1.53E-02
HCAR1	hydroxycarboxylic acid receptor 1	27198	1.743197098	1.53E-02
TACR2	tachykinin receptor 2	6865	1.52504192	1.53E-02
BTN3A3	butyrophilin, subfamily 3, member A3	10384	0.543680353	1.54E-02
IQCK	IQ motif containing K	124152	0.137836129	1.54E-02
PTMAP2	prothymosin, alpha pseudogene 2 (gene sequence 32)	5759	1.543418389	1.54E-02
RARG	retinoic acid receptor, gamma	5916	0.560176357	1.55E-02
DCN	decorin	1634	0.398058948	1.55E-02
EDA2R	ectodysplasin A2 receptor	60401	0.610792309	1.55E-02
RPL21P28	ribosomal protein L21 pseudogene 28	100131205	2.147450683	1.56E-02
NLRP10	NLR family, pyrin domain containing 10	338322	3.393551718	1.56E-02
CDH4	cadherin 4, type 1, R-cadherin (retinal)	1002	1.906601452	1.56E-02
LPAR1	lysophosphatidic acid receptor 1	1902	0.672145186	1.57E-02
DAAM1	dishevelled associated activator of morphogenesis 1	23002	0.51923459	1.58E-02
SPATA18	spermatogenesis associated 18	132671	0.449758982	1.58E-02
FYN	FYN oncogene related to SRC, FGR, YES	2534	0.597974181	1.58E-02
RGS5	regulator of G-protein signaling 5	8490	1.81519583	1.59E-02
SMC3	structural maintenance of chromosomes 3	9126	1.483944052	1.59E-02

Supplementary Table 1

TUBGCP5	tubulin, gamma complex associated protein 5	114791	1.564861852	1.60E-02
MLX	MAX-like protein X	6945	2.008942122	1.60E-02
ARHGAP18	Rho GTPase activating protein 18	93663	1.906576078	1.60E-02
SYN2	synapsin II	6854	0.325171788	1.60E-02
PLA2G4A	phospholipase A2, group IVA (cytosolic, calcium-dependent)	5321	0.544920964	1.63E-02
FTCD	formiminotransferase cyclodeaminase	10841	2.188673874	1.64E-02
PLK4	polo-like kinase 4	10733	1.825563055	1.64E-02
AMMECR1	Alport syndrome, mental retardation, midface hypoplasia and elliptocytosis chromosomal region gene 1	9949	0.690587583	1.64E-02
ENPP1	ectonucleotide pyrophosphatase/phosphodiesterase 1	5167	2.249895508	1.65E-02
QSOX2	quiescin Q6 sulfhydryl oxidase 2	169714	1.621315321	1.65E-02
BBIP1	BBSome interacting protein 1	92482	0.616871842	1.65E-02
CFI	complement factor I	3426	0.284872053	1.67E-02
NUPR1	nuclear protein, transcriptional regulator, 1	26471	0.523559824	1.68E-02
DENND5B-AS1	DENND5B antisense RNA 1	100874249	2.931520204	1.68E-02
TNK2	tyrosine kinase, non-receptor, 2	10188	1.860361256	1.68E-02
CERK	ceramide kinase	64781	1.626713519	1.68E-02
SLC44A1	solute carrier family 44, member 1	23446	0.602007222	1.68E-02
RNF41	ring finger protein 41	10193	0.623019101	1.69E-02
COL11A1	collagen, type XI, alpha 1	1301	3.355216823	1.70E-02

Supplementary Table 1

PLEKHG3	pleckstrin homology domain containing, family G (with RhoGef domain) member 3	26030	2.70128339	1.71E-02
KIF18B	kinesin family member 18B	146909	1.943861189	1.71E-02
SLC25A11	solute carrier family 25 (mitochondrial carrier; oxoglutarate carrier), member 11	8402	0.647382557	1.71E-02
CSGALNACT1	chondroitin sulfate N-acetylgalactosaminyltransferase 1	55790	0.511961597	1.73E-02
FBXO11	F-box protein 11	80204	1.530439591	1.73E-02
ZFP37	zinc finger protein 37 homolog (mouse)	7539	0.539798323	1.74E-02
HHIP-AS1	HHIP antisense RNA 1	646576	5.193891944	1.78E-02
KIAA2013	KIAA2013	90231	1.560059874	1.80E-02
MKL2	MKL/myocardin-like 2	57496	1.613574735	1.80E-02
MIR4253	microRNA 4253	100422914	0.349065336	1.81E-02
GSR	glutathione reductase	2936	0.665768815	1.83E-02
MYO10	myosin X	4651	2.037032503	1.84E-02
FASTK	Fas-activated serine/threonine kinase	10922	1.492538245	1.84E-02
GAB2	GRB2-associated binding protein 2	9846	0.611256921	1.85E-02
KLHL17	kelch-like 17 (Drosophila)	339451	1.853812843	1.87E-02
C14orf132	chromosome 14 open reading frame 132	56967	1.745284947	1.89E-02
FTL	ferritin, light polypeptide	2512	0.650879463	1.89E-02
SH2D4A	SH2 domain containing 4A	63898	0.510692968	1.90E-02
MCM3	minichromosome maintenance complex component 3	4172	1.789128612	1.91E-02
AOX1	aldehyde oxidase 1	316	0.525431755	1.91E-02

Supplementary Table 1

C11orf95	chromosome 11 open reading frame 95	65998	0.688210257	1.94E-02
AURKB	aurora kinase B	9212	1.884699479	1.95E-02
C1orf213	chromosome 1 open reading frame 213	148898	0.50953318	1.95E-02
GTPBP6	GTP binding protein 6 (putative)	8225	2.027042026	1.95E-02
ZNF671	zinc finger protein 671	79891	0.422731639	1.95E-02
ACSS1	acyl-CoA synthetase short-chain family member 1	84532	0.335761333	1.97E-02
STMN1	stathmin 1	3925	1.6413253	1.99E-02
RBBP8P1	retinoblastoma binding protein 8 pseudogene 1	100420552	0.559230829	2.00E-02
CDK5R1	cyclin-dependent kinase 5, regulatory subunit 1 (p35)	8851	2.777654192	2.01E-02
LRP12	low density lipoprotein receptor-related protein 12	29967	1.721773538	2.01E-02
PDCD4-AS1	PDCD4 antisense RNA 1	282997	0.601079241	2.01E-02
APRT	adenine phosphoribosyltransferase	353	2.449154863	2.02E-02
GPAM	glycerol-3-phosphate acyltransferase, mitochondrial	57678	3.376374656	2.05E-02
DUSP3	dual specificity phosphatase 3	1845	1.718240878	2.06E-02
IDE	insulin-degrading enzyme	3416	0.707713576	2.07E-02
PALM	paralemmin	5064	0.187614339	2.07E-02
PDLIM3	PDZ and LIM domain 3	27295	4.630185521	2.07E-02
RHBDF2	rhomboid 5 homolog 2 (Drosophila)	79651	5.353108892	2.07E-02
GPR157	G protein-coupled receptor 157	80045	1.956984596	2.07E-02
NCOA2	nuclear receptor coactivator 2	10499	0.652629995	2.07E-02

Supplementary Table 1

ZNF276	zinc finger protein 276	92822	1.795122254	2.07E-02
GSTK1	glutathione S-transferase kappa 1	373156	0.709746975	2.09E-02
TMEM154	transmembrane protein 154	201799	1.818751326	2.14E-02
DSN1	DSN1, MIND kinetochore complex component, homolog (S. cerevisiae)	79980	1.706138541	2.14E-02
ERF	Ets2 repressor factor	2077	1.679568225	2.14E-02
NAMPT	nicotinamide phosphoribosyltransferase	10135	1.497198492	2.14E-02
SVEP1	sushi, von Willebrand factor type A, EGF and pentraxin domain containing 1	79987	0.247102926	2.15E-02
LOX	lysyl oxidase	4015	0.466916022	2.16E-02
RFWD3	ring finger and WD repeat domain 3	55159	1.688632516	2.18E-02
ZNF575	zinc finger protein 575	284346	0.588936625	2.18E-02
YME1L1	YME1-like 1 (S. cerevisiae)	10730	1.465257173	2.19E-02
EIF4BP5	eukaryotic translation initiation factor 4B pseudogene 5	100421528	0.690034883	2.20E-02
TDP1	tyrosyl-DNA phosphodiesterase 1	55775	1.720661967	2.22E-02
KCNAB3	potassium voltage-gated channel, shaker-related subfamily, beta member 3	9196	1.426151849	2.22E-02
GSTO2	glutathione S-transferase omega 2	119391	2.378508202	2.24E-02
POLD1	polymerase (DNA directed), delta 1, catalytic subunit	5424	2.033986354	2.24E-02
ZNF181	zinc finger protein 181	339318	0.588339621	2.24E-02
YWHAQP6	YWHAQ pseudogene 6	100499253	1.611803644	2.24E-02
GINS3	GINS complex subunit 3 (Psf3 homolog)	64785	1.950961218	2.24E-02
THBS1	thrombospondin 1	7057	1.695654686	2.24E-02

Supplementary Table 1

ANGPTL4	angiopoietin-like 4	51129	3.778718376	2.25E-02
ST6GALNAC5	ST6 (alpha-N-acetyl-neuraminyl-2,3-beta-galactosyl-1,3)-N-acetylgalactosaminide alpha-2,6-sialyltransferase 5	81849	4.897606959	2.26E-02
SH3BP2	SH3-domain binding protein 2	6452	1.582941457	2.26E-02
PUS7	pseudouridylate synthase 7 homolog (S. cerevisiae)	54517	1.476476608	2.26E-02
RLF	rearranged L-myc fusion	6018	1.482795765	2.26E-02
RNF141	ring finger protein 141	50862	2.139644331	2.26E-02
CBX5	chromobox homolog 5	23468	1.508948847	2.27E-02
KIAA0513	KIAA0513	9764	0.545322807	2.27E-02
TFP1	transferrin pseudogene 1	100129696	1.597007845	2.27E-02
EDARADD	EDAR-associated death domain	128178	1.996893927	2.30E-02
UBA7	ubiquitin-like modifier activating enzyme 7	7318	0.542236337	2.30E-02
ATP5J2LP	ATP synthase, H+ transporting, mitochondrial Fo complex, subunit F2-like pseudogene	54100	1.864689087	2.30E-02
MT-ND3	mitochondrially encoded NADH dehydrogenase 3	4537	15.33685748	2.31E-02
SCO2	SCO cytochrome oxidase deficient homolog 2 (yeast)	9997	1.72294948	2.31E-02
CCDC82	coiled-coil domain containing 82	79780	1.666947653	2.32E-02
TOPBP1	topoisomerase (DNA) II binding protein 1	11073	1.612363467	2.32E-02
PLD5	phospholipase D family, member 5	200150	12.63610565	2.34E-02
ZSWIM4	zinc finger, SWIM-type containing 4	65249	2.741881079	2.34E-02

Supplementary Table 1

ZNF436	zinc finger protein 436	80818	0.511575259	2.34E-02
SIX2	SIX homeobox 2	10736	0.308996842	2.34E-02
NMI	N-myc (and STAT) interactor	9111	0.484483689	2.35E-02
PKIG	protein kinase (cAMP-dependent, catalytic) inhibitor gamma	11142	0.471679237	2.35E-02
CSMD2	CUB and Sushi multiple domains 2	114784	1.79451129	2.38E-02
SERTAD4-AS1	SERTAD4 antisense RNA 1	574036	0.312135207	2.38E-02
COX6B1P5	cytochrome c oxidase subunit VIb polypeptide 1 (ubiquitous) pseudogene 5	100289662	1.481256618	2.39E-02
MEG9	maternally expressed 9 (non-protein coding)	100507257	0.46647737	2.40E-02
MRPS33P2	mitochondrial ribosomal protein S33 pseudogene 2	359775	1.785444638	2.41E-02
SNX24	sorting nexin 24	28966	0.623942462	2.41E-02
HSD17B4	hydroxysteroid (17-beta) dehydrogenase 4	3295	0.693220248	2.41E-02
FAM227A	family with sequence similarity 227, member A	646851	0.686143529	2.41E-02
CDC14B	CDC14 cell division cycle 14 homolog B (S. cerevisiae)	8555	0.657313718	2.41E-02
LMLN-AS1	LMLN antisense RNA 1	100873947	1.504533156	2.41E-02
PCBD1	pterin-4 alpha-carbinolamine dehydratase/dimerization cofactor of hepatocyte nuclear factor 1 alpha	5092	0.601179825	2.41E-02
AEBP1	AE binding protein 1	165	1.683122055	2.43E-02
CHRNA1	cholinergic receptor, nicotinic, alpha 1 (muscle)	1134	10.96639926	2.45E-02
STAT2	signal transducer and activator of transcription 2, 113kDa	6773	0.618865499	2.46E-02
LPHN2	latrophilin 2	23266	0.503785813	2.46E-02

Supplementary Table 1

PCSK7	proprotein convertase subtilisin/kexin type 7	9159	1.900422773	2.47E-02
ASPA	aspartoacylase	443	0.194680731	2.53E-02
ARSI	arylsulfatase family, member I	340075	2.179599402	2.57E-02
TLE3	transducin-like enhancer of split 3 (E(sp1) homolog, Drosophila)	7090	1.494636646	2.58E-02
DUSP10	dual specificity phosphatase 10	11221	2.164894919	2.59E-02
UBALD2	UBA-like domain containing 2	283991	1.811497105	2.60E-02
DOK5	docking protein 5	55816	1.658857545	2.61E-02
BTN3A1	butyrophilin, subfamily 3, member A1	11119	0.555340762	2.63E-02
COL6A2	collagen, type VI, alpha 2	1292	2.131980141	2.64E-02
DZIP3	DAZ interacting protein 3, zinc finger	9666	1.770005692	2.66E-02
PPIL2	peptidylprolyl isomerase (cyclophilin)-like 2	23759	3.632568492	2.66E-02
SF3A3P2	splicing factor 3a, subunit 3 pseudogene 2	449645	2.8601702	2.66E-02
TIAM1	T-cell lymphoma invasion and metastasis 1	7074	0.665181938	2.66E-02
RGAG4	retrotransposon gag domain containing 4	340526	0.603034522	2.66E-02
GAS7	growth arrest-specific 7	8522	0.549641484	2.66E-02
POLR2J4	polymerase (RNA) II (DNA directed) polypeptide J4, pseudogene	84820	0.548679318	2.66E-02
USP53	ubiquitin specific peptidase 53	54532	0.508074443	2.66E-02
SPRED2	sprouty-related, EVH1 domain containing 2	200734	0.677052682	2.66E-02
LIPG	lipase, endothelial	9388	0.255588176	2.67E-02
UPP1	uridine phosphorylase 1	7378	1.707338952	2.68E-02

Supplementary Table 1

IMPDH1	IMP (inosine 5'-monophosphate) dehydrogenase 1	3614	1.517116737	2.68E-02
AHCTF1	AT hook containing transcription factor 1	25909	1.444301061	2.68E-02
CSRP1	cysteine and glycine-rich protein 1	1465	1.785116091	2.68E-02
PCSK9	proprotein convertase subtilisin/kexin type 9	255738	0.164963071	2.69E-02
CYB561	cytochrome b-561	1534	0.638594087	2.69E-02
PTPN12	protein tyrosine phosphatase, non-receptor type 12	5782	1.459985357	2.69E-02
EPHB1	EPH receptor B1	2047	3.693913287	2.76E-02
PRKDC	protein kinase, DNA-activated, catalytic polypeptide	5591	1.719063575	2.76E-02
SNX18	sorting nexin 18	112574	0.626941658	2.76E-02
JAG1	jagged 1	182	0.228252569	2.76E-02
KREMEN2	kringle containing transmembrane protein 2	79412	2.60341806	2.76E-02
PAM	peptidylglycine alpha-amidating monooxygenase	5066	1.486674179	2.79E-02
BLOC1S5-TXNDC5	BLOC1S5-TXNDC5 readthrough (non-protein coding)	100526836	0.247759623	2.80E-02
DFNA5	deafness, autosomal dominant 5	1687	0.593006408	2.82E-02
PTPRE	protein tyrosine phosphatase, receptor type, E	5791	2.469067025	2.84E-02
IL17RA	interleukin 17 receptor A	23765	1.623641252	2.84E-02
APOBEC3C	apolipoprotein B mRNA editing enzyme, catalytic polypeptide-like 3C	27350	0.679739563	2.84E-02
OXTR	oxytocin receptor	5021	5.371700987	2.86E-02
HAUS5	HAUS augmin-like complex, subunit 5	23354	1.645308251	2.86E-02

Supplementary Table 1

C5orf45	chromosome 5 open reading frame 45	51149	0.70369248	2.86E-02
TNFRSF10B	tumor necrosis factor receptor superfamily, member 10b	8795	1.510525354	2.87E-02
GMNN	geminin, DNA replication inhibitor	51053	1.829284414	2.88E-02
SNORA51	small nucleolar RNA, H/ACA box 51	677831	0.671930676	2.88E-02
ZNF66	zinc finger protein 66	7617	2.141616263	2.88E-02
AGAP1	ArfGAP with GTPase domain, ankyrin repeat and PH domain 1	116987	1.614060213	2.88E-02
NNT-AS1	NNT antisense RNA 1	100652772	0.66943496	2.88E-02
ITM2B	integral membrane protein 2B	9445	0.678425264	2.89E-02
NR4A3	nuclear receptor subfamily 4, group A, member 3	8013	4.285899439	2.90E-02
NBR1	neighbor of BRCA1 gene 1	4077	0.692657999	2.92E-02
CARD9	caspase recruitment domain family, member 9	64170	1.822710832	2.92E-02
PDE4B	phosphodiesterase 4B, cAMP-specific	5142	3.269624593	3.00E-02
MCAM	melanoma cell adhesion molecule	4162	4.367473653	3.02E-02
FLG-AS1	FLG antisense RNA 1	339400	0.1149781	3.02E-02
KIF20B	kinesin family member 20B	9585	1.71872985	3.03E-02
DAW1	dynein assembly factor with WDR repeat domains 1	164781	7.450317974	3.04E-02
RASSF1	Ras association (RalGDS/AF-6) domain family member 1	11186	1.461719816	3.04E-02
CIC	capicua homolog (Drosophila)	23152	1.675011267	3.05E-02
TSPAN13	tetraspanin 13	27075	3.160524503	3.08E-02
DDX39A	DEAD (Asp-Glu-Ala-Asp) box polypeptide 39A	10212	1.783007535	3.08E-02

Supplementary Table 1

TBC1D16	TBC1 domain family, member 16	125058	0.719251086	3.08E-02
KLF4	Kruppel-like factor 4 (gut)	9314	0.399699868	3.08E-02
METTL7A	methyltransferase like 7A	25840	0.363898245	3.08E-02
IDH2	isocitrate dehydrogenase 2 (NADP+), mitochondrial	3418	1.50288373	3.09E-02
MIR5683	microRNA 5683	100847034	5.293070978	3.10E-02
DNTTIP1	deoxynucleotidyltransferase, terminal, interacting protein 1	116092	1.671970624	3.10E-02
FOXK2	forkhead box K2	3607	1.415951451	3.10E-02
FTLP3	ferritin, light polypeptide pseudogene 3	284764	0.667895269	3.10E-02
CBFB	core-binding factor, beta subunit	865	1.486398448	3.13E-02
WDR76	WD repeat domain 76	79968	1.941094156	3.15E-02
PROB1	proline-rich basic protein 1	389333	4.340562452	3.19E-02
WDR34	WD repeat domain 34	89891	0.685941763	3.24E-02
TMEM258	transmembrane protein 258	746	1.836075648	3.25E-02
KIAA0895	KIAA0895	23366	1.788006399	3.25E-02
RAD51AP1	RAD51 associated protein 1	10635	1.778660051	3.25E-02
RNA5SP437	RNA, 5S ribosomal pseudogene 437	100873686	1.456971442	3.25E-02
MT1L	metallothionein 1L (gene/pseudogene)	4500	3.100235909	3.25E-02
DTWD2	DTW domain containing 2	285605	1.512862006	3.27E-02
HBEGF	heparin-binding EGF-like growth factor	1839	3.743440957	3.30E-02
SLC6A7	solute carrier family 6 (neurotransmitter transporter, L-proline), member 7	6534	2.182559523	3.32E-02

Supplementary Table 1

PHRF1	PHD and ring finger domains 1	57661	1.624637331	3.33E-02
SPR	sepiapterin reductase (7,8-dihydrobiopterin:NADP+ oxidoreductase)	6697	0.524803592	3.33E-02
PROCR	protein C receptor, endothelial	10544	0.661434682	3.34E-02
SLC29A1	solute carrier family 29 (nucleoside transporters), member 1	2030	2.026485707	3.35E-02
GPHA2	glycoprotein hormone alpha 2	170589	1.520837805	3.37E-02
SZT2	seizure threshold 2 homolog (mouse)	23334	1.824657517	3.38E-02
SERPINA1	serpin peptidase inhibitor, clade A (alpha-1 antiproteinase, antitrypsin), member 1	5265	9.893122904	3.44E-02
ARHGEF3	Rho guanine nucleotide exchange factor (GEF) 3	50650	0.479202387	3.44E-02
GPR17	G protein-coupled receptor 17	2840	0.473743811	3.44E-02
SMC2	structural maintenance of chromosomes 2	10592	1.617235973	3.49E-02
KIAA0930	KIAA0930	23313	0.657241097	3.49E-02
TSPAN2	tetraspanin 2	10100	0.222466653	3.49E-02
XRCC2	X-ray repair complementing defective repair in Chinese hamster cells 2	7516	1.90658969	3.54E-02
DBF4	DBF4 homolog (S. cerevisiae)	10926	1.480028749	3.57E-02
ZMYND8	zinc finger, MYND-type containing 8	23613	1.432299601	3.57E-02
LMF2	lipase maturation factor 2	91289	1.788438985	3.59E-02
HELLS	helicase, lymphoid-specific	3070	1.768074787	3.59E-02
PCDH18	protocadherin 18	54510	0.538415445	3.59E-02
ZHX2	zinc fingers and homeoboxes 2	22882	0.464723337	3.61E-02
PLIN2	perilipin 2	123	1.937997756	3.62E-02

Supplementary Table 1

TRPC1	transient receptor potential cation channel, subfamily C, member 1	7220	1.616901843	3.62E-02
WDFY2	WD repeat and FYVE domain containing 2	115825	1.47740821	3.62E-02
SOCS5	suppressor of cytokine signaling 5	9655	0.638625206	3.62E-02
ZMAT3	zinc finger, matrin-type 3	64393	0.635244155	3.62E-02
MCC	mutated in colorectal cancers	4163	0.601730254	3.62E-02
OLFML2A	olfactomedin-like 2A	169611	0.363264175	3.62E-02
CDKN2D	cyclin-dependent kinase inhibitor 2D (p19, inhibits CDK4)	1032	2.029226632	3.63E-02
KIAA1841	KIAA1841	84542	0.647760499	3.71E-02
RAI14	retinoic acid induced 14	26064	1.521785794	3.72E-02
PFN1P10	profilin 1 pseudogene 10	767853	0.586606922	3.72E-02
FSIP1	fibrous sheath interacting protein 1	161835	1.654844548	3.74E-02
ADAP2	ArfGAP with dual PH domains 2	55803	0.692953572	3.74E-02
STRIP2	striatin interacting protein 2	57464	2.263377145	3.79E-02
GRIP2	glutamate receptor interacting protein 2	80852	1.947295327	3.79E-02
GTF2E1	general transcription factor IIE, polypeptide 1, alpha 56kDa	2960	1.461375069	3.82E-02
NT5DC4	5'-nucleotidase domain containing 4	284958	1.74635529	3.84E-02
FAM73A	family with sequence similarity 73, member A	374986	1.412732188	3.84E-02
URGCP	upregulator of cell proliferation	55665	0.614004443	3.84E-02
EPS8	epidermal growth factor receptor pathway substrate 8	2059	0.729792131	3.86E-02
SNORA43	small nucleolar RNA, H/ACA box 43	677824	1.639702861	3.87E-02

Supplementary Table 1

MELK	maternal embryonic leucine zipper kinase	9833	1.769179708	3.89E-02
TUFT1	tuftelin 1	7286	0.466393957	3.89E-02
MIR503HG	MIR503 host gene	84848	0.493097553	3.91E-02
PMP22	peripheral myelin protein 22	5376	0.588529403	3.93E-02
ST7L	suppression of tumorigenicity 7 like	54879	0.518225783	3.93E-02
CXCL5	chemokine (C-X-C motif) ligand 5	6374	4.277375905	3.93E-02
IPMK	inositol polyphosphate multikinase	253430	1.551086238	3.93E-02
PRKAR1A	protein kinase, cAMP-dependent, regulatory, type I, alpha	5573	0.375677079	3.95E-02
LRRFIP1	leucine rich repeat (in FLII) interacting protein 1	9208	1.594455131	3.97E-02
CCNA2	cyclin A2	890	1.501554355	3.97E-02
WNT5B	wingless-type MMTV integration site family, member 5B	81029	2.594876721	3.99E-02
DNAH3	dynein, axonemal, heavy chain 3	55567	0.568332373	4.00E-02
SORT1	sortilin 1	6272	1.662441184	4.01E-02
PRDX2P1	peroxiredoxin 2 pseudogene 1	359844	1.502181775	4.06E-02
STARD4-AS1	STARD4 antisense RNA 1	100505678	0.676129641	4.06E-02
DOCK7	dedicator of cytokinesis 7	85440	1.520464887	4.08E-02
DCHS2	dachsous 2 (Drosophila)	54798	2.461500444	4.10E-02
SNORA75	small nucleolar RNA, H/ACA box 75	654321	1.726481171	4.10E-02
WDR81	WD repeat domain 81	124997	0.491465823	4.10E-02
ITGB5-AS1	ITGB5 antisense RNA 1	100873992	0.726323971	4.11E-02

Supplementary Table 1

C4orf19	chromosome 4 open reading frame 19	55286	1.826182059	4.13E-02
TRPC4	transient receptor potential cation channel, subfamily C, member 4	7223	3.548272968	4.15E-02
CEP57L1	centrosomal protein 57kDa-like 1	285753	1.422861758	4.20E-02
CSNK1E	casein kinase 1, epsilon	1454	1.397661213	4.23E-02
CCDC138	coiled-coil domain containing 138	165055	1.825069748	4.24E-02
HAUS6	HAUS augmin-like complex, subunit 6	54801	1.671773503	4.25E-02
TXN2	thioredoxin 2	25828	0.718939213	4.26E-02
MMP8	matrix metallopeptidase 8 (neutrophil collagenase)	4317	4.78766099	4.26E-02
COA3	cytochrome C oxidase assembly factor 3	28958	3.12861037	4.26E-02
SPTAN1	spectrin, alpha, non-erythrocytic 1	6709	0.696540237	4.26E-02
CORO6	coronin 6	84940	0.56019418	4.26E-02
NAMPTL	nicotinamide phosphoribosyltransferase-like	646309	1.507146543	4.27E-02
CAMK2A	calcium/calmodulin-dependent protein kinase II alpha	815	2.181183278	4.28E-02
PODNL1	podocan-like 1	79883	1.734280552	4.28E-02
SERPINH1	serpin peptidase inhibitor, clade H (heat shock protein 47), member 1, (collagen binding protein 1)	871	0.61617519	4.30E-02
SH3BP5-AS1	SH3BP5 antisense RNA 1	100505696	0.649785475	4.31E-02
VEGFB	vascular endothelial growth factor B	7423	1.503360288	4.34E-02
CDK1	cyclin-dependent kinase 1	983	1.745610639	4.41E-02
RAVER1	ribonucleoprotein, PTB-binding 1	125950	1.45261401	4.41E-02

Supplementary Table 1

DPF2	D4, zinc and double PHD fingers family 2	5977	0.712992497	4.41E-02
HGF	hepatocyte growth factor (hepapoietin A; scatter factor)	3082	0.251959368	4.41E-02
ZNHIT3	zinc finger, HIT-type containing 3	9326	1.754978796	4.43E-02
MROH2A	maestro heat-like repeat family member 2A	339766	1.952610373	4.44E-02
KDM4A-AS1	KDM4A antisense RNA 1	100132774	0.729614851	4.45E-02
MTERFD2	MTERF domain containing 2	130916	0.367263136	4.48E-02
VWA8	von Willebrand factor A domain containing 8	23078	2.183252512	4.51E-02
PTMA	prothymosin, alpha	5757	1.451946907	4.51E-02
MMACHC	methylmalonic aciduria (cobalamin deficiency) cblC type, with homocystinuria	25974	0.733575469	4.51E-02
CILP	cartilage intermediate layer protein, nucleotide pyrophosphohydrolase	8483	0.311590899	4.51E-02
RIF1	RAP1 interacting factor homolog (yeast)	55183	1.423531187	4.53E-02
SMS	spermine synthase	6611	1.624637218	4.53E-02
CHN2	chimerin (chimaerin) 2	1124	0.309279904	4.60E-02
UBE2C	ubiquitin-conjugating enzyme E2C	11065	1.847270578	4.63E-02
KDM4B	lysine (K)-specific demethylase 4B	23030	1.681460281	4.63E-02
PGK1P2	phosphoglycerate kinase 1, pseudogene 2	5233	2.285942318	4.68E-02
PTPN13	protein tyrosine phosphatase, non-receptor type 13 (APO-1/CD95 (Fas)-associated phosphatase)	5783	0.672199791	4.69E-02
FBXO5	F-box protein 5	26271	1.625155538	4.69E-02
CASK-AS1	CASK antisense RNA 1	100873928	1.396405844	4.74E-02

Supplementary Table 1

DNAJC22	DnaJ (Hsp40) homolog, subfamily C, member 22	79962	4.114254389	4.75E-02
ARPP19	cAMP-regulated phosphoprotein, 19kDa	10776	1.395816315	4.75E-02
BCKDHB	branched chain keto acid dehydrogenase E1, beta polypeptide	594	0.647257373	4.75E-02
BNC1	basonuclin 1	646	1.619873552	4.76E-02
SNRPN	small nuclear ribonucleoprotein polypeptide N	6638	0.663935363	4.76E-02
RHOD	ras homolog family member D	29984	0.453233059	4.76E-02
CDC42EP2	CDC42 effector protein (Rho GTPase binding) 2	10435	0.572782055	4.79E-02
SOGA1	suppressor of glucose, autophagy associated 1	140710	1.435662663	4.80E-02
ADCY9	adenylate cyclase 9	115	0.681951763	4.80E-02
BARD1	BRCA1 associated RING domain 1	580	1.802217084	4.81E-02
RALGPS2	Ral GEF with PH domain and SH3 binding motif 2	55103	0.565929325	4.82E-02
GTSE1	G-2 and S-phase expressed 1	51512	1.70806846	4.83E-02
MYO1B	myosin IB	4430	0.644854731	4.84E-02
EXO1	exonuclease 1	9156	1.906814895	4.90E-02
ALDH1A1	aldehyde dehydrogenase 1 family, member A1	216	0.066772136	4.92E-02
GTF2H5	general transcription factor IIH, polypeptide 5	404672	0.587626352	4.96E-02
ZNRF3	zinc and ring finger 3	84133	1.882232832	4.96E-02
SLC26A6	solute carrier family 26, member 6	65010	1.719368881	4.96E-02
ZNF738	zinc finger protein 738	148203	1.577746803	4.96E-02
PROS1	protein S (alpha)	5627	0.620140493	4.96E-02

Supplementary Table 1

AP4B1	adaptor-related protein complex 4, beta 1 subunit	10717	1.678140025	4.98E-02

Table S1. List of differentially expressed genes between ADSC expanded under 21% and 1% oxygen. Gene symbols, gene names, EntrezGene ID and expression fold change (21%/1%) are given. Genes are listed in order of statistical significance based on q-values (Benjamini & Hochberg adjusted).

Supplementary Table 2

KEGG Pathways	Number of reference genes	Number of genes in the gene set	Expected number in the category	Ratio of enrichment	q-valuE (BH)
Metabolic pathways	1130	65	19.57	3.32	6.22E-15
Glutathione metabolism	50	13	0.87	15.01	1.53E-10
Purine metabolism	162	15	2.81	5.35	7.81E-06
Cell cycle	124	13	2.15	6.05	9.18E-06
Glycolysis / Gluconeogenesis	65	9	1.13	7.99	4.93E-05
Pentose phosphate pathway	27	6	0.47	12.83	0.0001
Metabolism of xenobiotics by cytochrome P450	71	8	1.23	6.51	0.0006
Drug metabolism - cytochrome P450	73	8	1.26	6.33	0.0007
Calcium signaling pathway	177	12	3.07	3.91	0.0009
Pathways in cancer	326	17	5.65	3.01	0.0009
p53 signaling pathway	68	7	1.18	5.94	0.0025
Parkinson's disease	130	9	2.25	4.00	0.0057
TGF-beta signaling pathway	84	7	1.45	4.81	0.0063
Nicotinate and nicotinamide metabolism	24	4	0.42	9.62	0.0069
Glycosaminoglycan biosynthesis - heparan sulfate	26	4	0.45	8.88	0.0086
Nucleotide excision repair	44	5	0.76	6.56	0.0086
Complement and coagulation cascades	69	6	1.20	5.02	0.0105
Renal cell carcinoma	70	6	1.21	4.95	0.0107
Pyrimidine metabolism	99	7	1.71	4.08	0.0123
Glycosphingolipid biosynthesis - ganglio series	15	3	0.26	11.55	0.0137
Glycine, serine and threonine metabolism	32	4	0.55	7.22	0.0144
Cytokine-cytokine receptor interaction	265	12	4.59	2.61	0.0156
Focal adhesion	200	10	3.46	2.89	0.0161
Protein digestion and absorption	81	6	1.40	4.28	0.0166
Oocyte meiosis	112	7	1.94	3.61	0.0170
Cysteine and methionine metabolism	36	4	0.62	6.41	0.0170

Supplementary Table 2

ECM-receptor interaction	85	6	1.47	4.08	0.0170
DNA replication	36	4	0.62	6.41	0.0170
One carbon pool by folate	18	3	0.31	9.62	0.0170
Vitamin B6 metabolism	6	2	0.10	19.24	0.0196
Bladder cancer	42	4	0.73	5.50	0.0261
Osteoclast differentiation	128	7	2.22	3.16	0.0286
Mismatch repair	23	3	0.40	7.53	0.0286
Nitrogen metabolism	23	3	0.40	7.53	0.0286
MAPK signaling pathway	268	11	4.64	2.37	0.0294
Oxidative phosphorylation	132	7	2.29	3.06	0.0316
Cell adhesion molecules (CAMs)	133	7	2.30	3.04	0.0322
Arrhythmogenic right ventricular cardiomyopathy (ARVC)	74	5	1.28	3.90	0.0334
Amino sugar and nucleotide sugar metabolism	48	4	0.83	4.81	0.0334
Homologous recombination	28	3	0.48	6.19	0.0411
Fc epsilon RI signaling pathway	79	5	1.37	3.65	0.0411
Histidine metabolism	29	3	0.50	5.97	0.0440
Arginine and proline metabolism	54	4	0.94	4.28	0.0445
Starch and sucrose metabolism	54	4	0.94	4.28	0.0445
Hypertrophic cardiomyopathy (HCM)	83	5	1.44	3.48	0.0451
Hedgehog signaling pathway	56	4	0.97	4.12	0.0469
Wnt signaling pathway	150	7	2.60	2.69	0.0469
Progesterone-mediated oocyte maturation	86	5	1.49	3.36	0.0485
Arachidonic acid metabolism	59	4	1.02	3.91	0.0496
Base excision repair	33	3	0.57	5.25	0.0496
Jak-STAT signaling pathway	155	7	2.68	2.61	0.0496
Lysosome	121	6	2.10	2.86	0.0496
Apoptosis	87	5	1.51	3.32	0.0496

Table S2. Complete list of enriched pathways from differentially expressed genes between ADSC cultured under 21% or 1% oxygen. Gene symbols, the number of reference genes in the KEGG category, the number of genes in the genes set of differentially expressed gene list, the expected number in the category and the ratio of enrichment are given. Pathways are listed in order of statistical significance based on q-values (Benjamini & Hochberg adjusted).
